# Conduction Block in the Human Ischemic Myocardium: Insights from a 1D Electromechanical Model

**DOI:** 10.3390/ijms27146302

**Published:** 2026-07-15

**Authors:** Alexander Kursanov, Nathalie A. Balakina-Vikulova, Olga Solovyova, Leonid B. Katsnelson

**Affiliations:** 1 Laboratory of Mathematical Physiology, Institute of Immunology and Physiology, Ural Branch of the Russian Academy of Sciences, 620049 Ekaterinburg, Russia; 2 Institute of Natural Sciences and Mathematics, Ural Federal University, 620002 Ekaterinburg, Russia

**Keywords:** myocardium, electromechanical cellular and tissue models, ischemia, conduction block, gap junction, sodium currents

## Abstract

Acute myocardial ischemia, caused by a sudden reduction in coronary blood flow, initiates metabolic disturbances that lead to severe pathophysiological consequences. These include electrophysiological alterations, such as changes in action potential morphology and impaired electrotonic coupling between cardiomyocytes, and mechanical dysfunction, characterized by reduced contractile force and subsequent mechanical discoordination across the ventricular wall. This study employs multi-scale mathematical modeling to investigate the effects of acute ischemia on the electromechanical activity of a single human cardiomyocyte and a one-dimensional myocardial tissue. We identify the conditions for conduction block initiation and the parameters governing conduction restoration in ischemic tissue, and analyze the underlying mechanisms. Our simulations demonstrate that conduction slowing in the one-dimensional strand under ischemia directly results from the hyperkalemia-induced reduction in the fast sodium current (*i_Na_*). This *i_Na_* reduction is enhanced by direct electromechanical coupling and mechano-electric/mechano-calcium feedback in the mechanically and electrically interacting cardiomyocytes of the one-dimensional tissue. Under 15 min ischemia conditions, *i_Na_* decreases to a level insufficient to sustain excitation propagation, causing conduction block. Under the conditions of this simulation, where gap junction conductance was held unchanged, the block occurred via the *i_Na_* reduction which is itself amplified by mechano-calcium feedback. Furthermore, our model suggests a potential compensatory mechanism against conduction block in ischemic myocardium. Experimental evidence indicates that ischemia can disrupt gap junctions. A moderate reduction in the electrodiffusion coefficient along the strand, simulating reduced gap junction conductance, can convert persistent conduction block into a transient form and even eliminate it completely, facilitating the maintenance of excitation wave propagation.

## 1. Introduction

Ischemic heart disease remains a leading cause of sudden cardiac death worldwide. An acute ischemic event, characterized by coronary artery obstruction, severely compromises oxygen delivery to the myocardium. This initiates a pathological cascade of metabolic disturbances, primarily hypoxia, extracellular hyperkalemia (elevated [K^+^]*_o_*), intracellular ATP depletion, and acidosis. These metabolic alterations, in turn, trigger profound electrophysiological dysfunction, including changes in action potential duration, slowed conduction velocity, and the development of conduction blocks, which create a substrate for lethal arrhythmias such as ventricular fibrillation [1,2,3].

Furthermore, acute ischemia critically impairs the mechanical function of the heart. The resulting systolic and diastolic dysfunction arises from the interplay of several mechanisms. Crucially, electrophysiological abnormalities, and particularly action potential shortening, impair the kinetics of cytosolic Ca^2+^ handling, thereby compromising a key process in force generation [1,2,4]. Furthermore, ATP depletion within the myofilaments directly reduces the number of force-generating cross-bridges, further diminishing contractile force. Ultimately, the disruption of electromechanical coupling and the increased tissue heterogeneity caused by emerging ischemic zones lead to a discoordination of contraction across the myocardium, severely depressing the heart’s overall pump function.

This study utilizes a multi-scale computational approach to simulate and analyze the effects of acute ischemia on cardiac function. We employ a human ventricular cardiomyocyte model to examine changes in action potential morphology, calcium transients, and force generation under ischemic conditions. This model is then incorporated into a one-dimensional (1D) tissue strand to investigate the resulting alterations in excitation propagation and force generation at the macroscopic level. A key focus is on identifying how specific ischemic factors, such as hyperkalemia, diminished fast sodium current and disruption of gap junction, affect conduction velocity and the initiation of conduction block.

The contribution of these factors to conduction block has been analyzed previously, but primarily using purely electrophysiological mathematical models [5,6,7,8]. In these works, all the listed factors were correctly considered to cause slowing of the excitation wave in myocardial tissue and for this reason, all of them were a priori presumed as more or less significant preconditions for the conduction block. The authors of these detailed studies convincingly demonstrated that the slowing of the excitation wave caused by the disruption of gap junctions contributes the least to conduction block compared to the other factors. However, none of these studies analyzed the possibility that this particular factor of the wave slowing (gap junction partial disruption) under certain conditions could oppose all the other factors listed above, i.e., not only make a small contribution to the block, but even, to some extent, prevent its occurrence during the acute ischemia. Meanwhile, in 2006 Dhein did propose such a hypothesis [9]. Our assessment of this seemingly counterintuitive assumption using the mathematical model confirms its validity.

Additionally, our previous modeling has demonstrated that mechanical activity of the cardiomyocytes and mechano-electric/mechano-calcium feedback loops can substantially modulate pathological effects in the myocardium [10]. Therefore, we consider it essential to employ electromechanical models that incorporate such feedback loops in our analysis of the occurrence and overcoming of the conduction block, presented in this study. The results presented here indicate that mechanical interaction between the cardiomyocytes in the myocardial tissue, jointly with the intracellular mechano-electric/mechano-calcium feedback loops, may significantly affect the occurrence of electrical excitation conduction block during acute ischemia.

## 2. Results

In this study, mathematical modeling of the electromechanical coupling during acute myocardial ischemia was performed both at the level of the cardiomyocyte model and that of one-dimensional myocardial tissue (see Section 4 below for details). In particular, we introduce two 1D models of ischemia: model ***IM*1_15_**, in which the electrodiffusion coefficient is kept unchanged relative to healthy myocardium, and model ***IM*2_15_**, in which this parameter is reduced.

*Simulation of single cardiomyocyte and 1D tissue electrical and mechanical activity during ischemia development*

**Figure 1:**
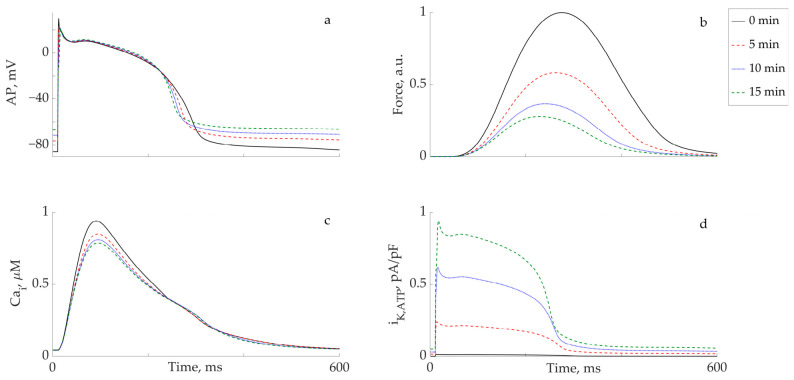
Changes during ischemia at 0, 5, 10, and 15 min of the acute ischemia in simulated isometric contractions of a single cell. (**a**) Time course of action potential (AP) generation; (**b**) active force generated by the cardiomyocyte; (**c**) intracellular Ca^2+^ concentration (Ca_i_); (**d**) ATP-sensitive potassium current (*i_K_*_,*ATP*_). All presented signals are steady states for the corresponding stage of ischemia. Force in panel (**b**) is normalized to the peak value in the healthy myocyte (0 min).

**Figure 2:**
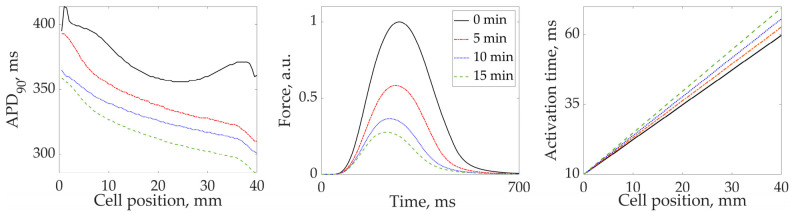
Changes during ischemia at 0, 5, 10, and 15 min of the acute ischemia in the 1D strand simulation. (**Left**) Changes in action potential duration at 90% repolarization level (APD_90_) in cells along the strand. (**Middle**) Active force generated by the strand in isometric contractions (normalized to its peak value for the healthy state). (**Right**) Arrival time of the depolarization wave to each cell. The traces for the 15 min ischemia correspond to the first 18 stimuli following the onset of this ischemic phase. Subsequently, a conduction block occurred.

Note that the 1D strand in our simulations consists of identical cardiomyocytes, the same as those used in the single-cell simulations. Nevertheless, due to the finite velocity of propagation of the excitation wave in the 1D tissue from *x* = 0 toward *x = x_F_*, a delay appears in the pacing of the cells, which is greater the longer the distance of a particular cell from the edge of the strand (*x* = 0) to which the electrical stimulus is applied. The resulting asynchrony of the mechanical response of cells along the strand, combined with continuous mechanical interaction between all these cells, leads to a reduction in the active force of each cardiomyocyte. As a result, the force generated by the strand (which coincides with that of individual cells) is always lower than under isolated conditions. To ensure that this difference does not cloud the assessment of the contribution of ischemia to force development, we eliminate it by showing the force in both the single-cell and the strand simulations (Figure 1 and Figure 2) normalized to their respective healthy controls: to the isolated myocyte for Figure 1 and to the healthy tissue for Figure 2.

In general, Figure 1 reveals electrical and mechanical signals and Ca^2+^ transients in simulations of isometric contractions of the isolated cardiomyocyte at various stages of ischemia (5, 10, and 15 min of ischemia) compared to the 0 min condition (i.e., the healthy cardiomyocyte). In Figure 2, we present the results of numerical experiments for the 1D homogeneous strand formed of the same cardiomyocytes at the same stages. The 15 min ischemia in Figure 2 is represented by the 1D ***IM*1_15_** model, in which the electrodiffusion coefficient *D* remains unchanged compared to the 1D model of the healthy myocardium. In other words, the 15 min ischemia in this simulation is provided only by the intracellular ischemic changes.

Ischemia is characterized by a pronounced shortening of the action potential and elevated diastolic depolarization of the cell membrane, resulting in severely impaired cardiomyocyte contractility.

The way hyperkalemia alters the resting and action potentials follows well-characterized, nonlinear [K^+^]*_o_*-dependencies established in earlier modeling work [11]. Elevated [K^+^]*_o_* depolarizes the resting potential through the shift in the K^+^ equilibrium potential; this depolarization raises the resting inactivation of the fast Na^+^ channels—decreasing *i_Na_* availability even without any change in *g_Na_*—whereas the [K^+^]*_o_*-dependent increase in the inward-rectifier (*i_K_*_1_) and rapid delayed-rectifier (*i_Kr_*) conductances promotes AP shortening [12]. The same [K^+^]*_o_*-dependent mechanisms, in their opposite (hypokalemic) direction, have been reviewed and illustrated for the human ventricle by Trenor and colleagues [13], as well as within a broader modeling framework for K^+^- and drug-dependent human ventricular electrophysiology [14].

In full agreement with numerous experimental and theoretical results, hyperkalemia causes a significant depolarization of the resting potential (RP) in our simulations of an ischemic isolated cardiomyocyte presented in Figure 1c. RP = −85 mV for the healthy myocyte, while it is −76 mV during the 5 min ischemia, −70 mV during the 10 min ischemia, and −65 mV during the 15 min ischemia simulated in the same virtual single cardiomyocyte.

A decrease in the action potential (AP) duration during the development of acute ischemia in this single cell was also observed in our simulations. In particular, APD_90_ (AP duration measured at 90% repolarization) decreases from 328 ms for the healthy state to 275 ms during the 5 min ischemia, 260 ms during the 10 min ischemia, and 250 ms during the 15 min ischemia. This action-potential shortening is driven by the progressive activation of the ATP-sensitive potassium current (*i_K_*_,*ATP*_) with the ischemic stage, shown in Figure 1d: as [*ATP*]*_i_* falls, *i_K_*_,*ATP*_ grows from stage to stage, adding an outward K^+^ current that accelerates repolarization. According to published experimental data, species-specific features of the effect of ischemia/hypoxia on the AP duration are quite substantial. Significant effects of ischemia/hypoxia on APD_90_ have been reported for guinea pig myocardium, where AP duration has been shown to decrease several-fold as acute ischemia progresses [15]. Our previous simulations of ischemia using a mathematical model of electromechanical coupling in the guinea pig cardiomyocytes (*EO* model) did demonstrate similar pronounced effect [16]. Effects of ischemia on APD_90_, observed in other animals (e.g., rats [17], pigs [18], and cats [19]) were not as significant. Experimental estimates of the AP duration during ischemia in human myocardium are extremely limited. However, the available data [20] suggest that the 15–25% changes in APD_90_ obtained in our model are realistic. It is noteworthy that when we additionally accounted for the effect of reduced L-type calcium current (*i_CaL_*) due to intracellular acidosis by progressively decreasing its maximal conductance by 10% at 5 min, 30% at 10 min, and 50% at 15 min of acute ischemia using the approach proposed in the Shaw & Rudy simulations [11], we observed an even more pronounced impact of ischemia on the AP duration. However, in the study, we purposely ignore the effects of acidosis to focus on the intracellular mechanisms caused by hypoxia and hyperkalemia; therefore, the data on the reduced *i_CaL_* are not represented here.

Due to the mechanisms of electromechanical coupling, the duration of AP determines changes in the concentration of free Ca^2+^ in the cytosol (Ca^2+^ transient). As shown in Figure 1c, which illustrates Ca^2+^ transients during isometric contractions under normal conditions and at various stages of ischemia development, the amplitude of the Ca^2+^ transient decreases progressively with the duration of the ischemic episode. The decrease in Ca^2+^ amplitude during ischemia leads, in the model, to a decrease in the number of Ca^2+^ complexes with the regulatory protein troponin C. Consequently, this results in a significant reduction in the active force peak, the time to peak force, and the duration of isometric mechanical twitch in both cardiomyocyte and 1D strand simulations (Figure 1 and Figure 2).

Already at the 5th minute of ischemia, the active isometric peak force decreases by 1.5 times, and the time to the peak decreases by almost 5%. At 15 min of ischemia, the peak force is only 27% of the respective value in the healthy conditions, which indicates a significant mechanical dysfunction of cardiomyocytes both in the isolation and in the tissue under conditions of acute ischemia.

A common tendency is observed in both healthy and ischemic homogeneous myocardium to shorten the AP duration along the excitation wave propagation, despite the identity of all the cells composing the strand. Moreover, the action potential in each individual cell shortens significantly, as ischemia develops (Figure 2, left panel).

Excitation wave propagation along the strand slows down with the development of ischemia, despite the electrodiffusion coefficient *D* remaining unchanged in this simulation at all stages of ischemia from five to fifteen minutes (Figure 2, right panel). Specifically, conduction velocity along the 1D strand decreased from 0.8 m/s in healthy tissue to 0.75 m/s at 5 min of acute ischemia, 0.71 m/s at 10 min, and 0.67 m/s at 15 min, immediately before conduction block occurred in the latter case (Table 1). This trend is consistent with experimental observations of slowed conduction along the ischemic area of the ventricular myocardium.

**Figure 3:**
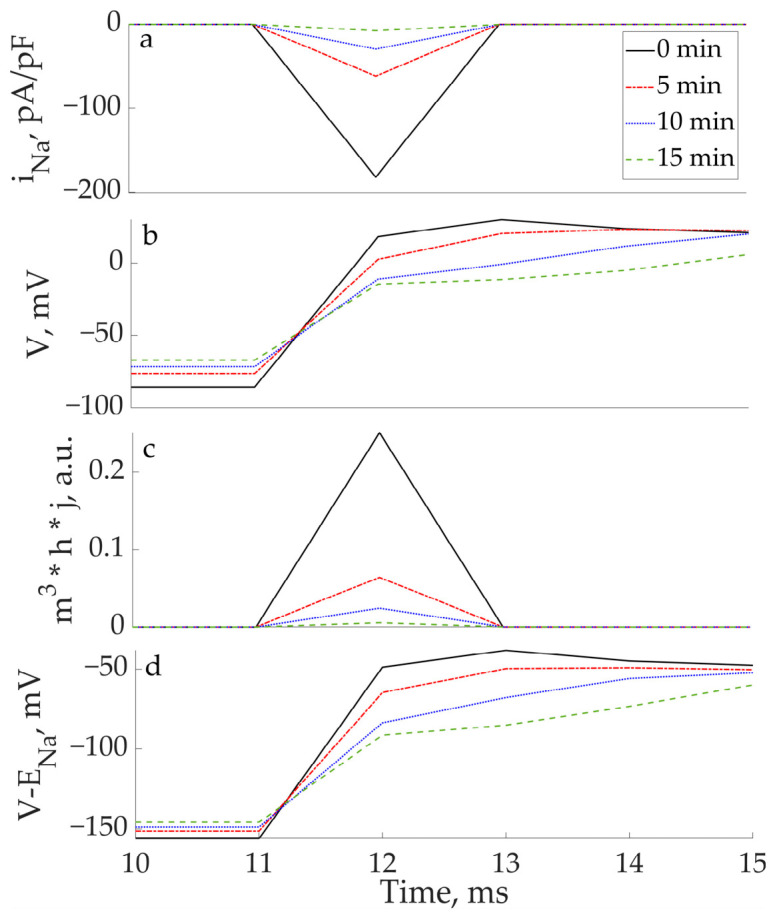
Time course of the fast Na^+^ current *i_Na_* (**a**) and its components according to Equation (5) at 0 (solid lines), 5 (dash-dotted lines), 10 (dotted lines), and 15 (dashed lines) minutes of the acute ischemia during simulated isometric contractions of a single cell within the first 5 ms after stimulus, when *i_Na_* is active. *V* represents the action potential (**b**); *m*, *h* and *j* are voltage-dependent gating variables of *i_Na_* (**c**); and *V*-*E_Na_* is the driving force for Na^+^ (**d**).

This effect is explained by a significant decrease in the fast sodium current *i_Na_* even in an isolated cardiomyocyte during the progression of acute ischemia, as shown in the corresponding panel of Figure 3a. It is important to emphasize that this current decreased markedly despite the fact that, during the ischemia simulation, neither the parameters of the voltage-dependent gating variables *m*, *h* and *j* for *i_Na_* nor the value of its maximal conductance *g_Na_* (Equation (5)) were altered.

**Table 1:** Influence of ischemia duration and electrodiffusion coefficient on conduction block patterns and safety factor in the simulated 1D tissue.

Stages of the Acute Ischemia, min	Electrodiffusion Coefficient (D), mm^2^/s	Conduction Velocity (m/s)	Block (Yes/No)	Safety Factor (SF)
0	300	0.8	no	1.54
5	300	0.75	no	1.28
10	300	0.71	no	1.16
15	300	0.67	yes	0.85
15	200	0.6	yes (transient) ^1^	0.93 (1.11) ^2^
15	150	0.4	no	1.15
15	<150	-	yes	

^1^ Conduction velocity was measured for the 18th stimulus (Figure 4, left panel). ^2^ Conduction block occurred transiently (every 2–3 stimuli); the values for conduction velocity and SF in parentheses refer to no-block conditions (Figure 4, middle panel).

The decrease in the amplitude of the *i_Na_* in ischemic cardiomyocytes in this case is mediated by the depolarization of the resting potential, which in turn is a consequence of the increase in the concentration of extracellular K^+^ during ischemia. This depolarization of the RP predetermines the initial rates of activation and inactivation of the Na^+^ channel gates. The subsequent voltage-dependent dynamics of these gates are determined by the interplay between the fast Na^+^ current *i_Na_* and the membrane potential during the first milliseconds after the stimulus. Over this interval, the two completely determine each other. This determines the activation of the Na^+^ gate. At the 5th minute after ischemia onset, the product “*m*^3^⋅*h*⋅*j*” is approximately five times lower than in the healthy cell; by the 15th minute, it is already two orders of magnitude lower (Figure 3c). This change in Na^+^ channel activation, according to Equation (5), is the main cause of the huge difference in the amplitude of the fast sodium current *i_Na_* observed at different stages of ischemia (Figure 3a).

**Figure 4:**
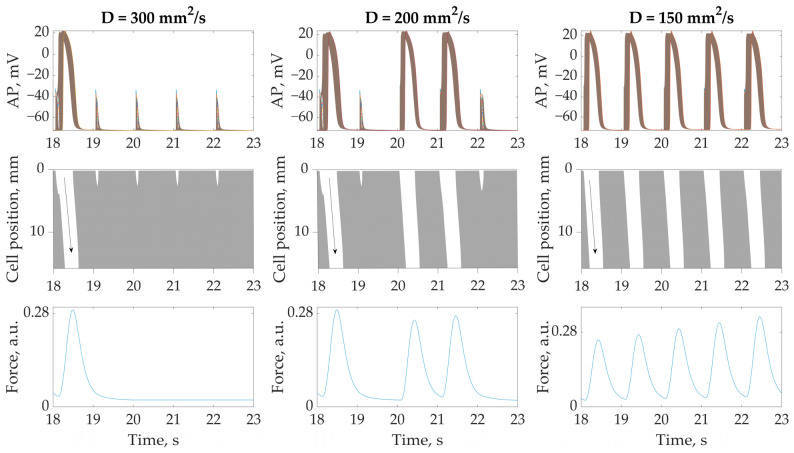
Occurrence of excitation conduction block in a 1D myocardial model at 15 min of ischemia is shown under three conditions: without reduction in the diffusion coefficient (***IM*1_15_** model) and with the diffusion coefficient *D* reduced by factors of 1.5 and 2 (***IM*2_15_** models), from left to right. The panels display events occurring between the 18th and 23rd simulated stimuli. (**Upper panels**) Membrane potential traces for cardiomyocytes within the 1D strand. Depending on the model used and the coefficient *D*, some stimuli trigger action potentials, whereas some stimuli elicit only short-lived, low-amplitude spikes that are incapable of triggering an action potential. These transient depolarizations are similar to the electrotonic responses of non-excitable tissue to external electrical stimulation. (**Middle panels**) Membrane potential along the 1D strand. The vertical axis shows the position along the strand’s length. In this representation, the strand is shown in its unstretched state, where each point on the axis corresponds to a single cell. The horizontal axis represents time. Regions where the membrane potential is above the resting potential are shown in white, while gray indicates quiescent tissue. Excitation is initiated at the top edge. The direction of propagation is indicated by an arrow. Complete white bands reveal successful propagation from top to bottom, whereas the abrupt cessation of white areas along the strand indicates that the block prevents the electrical signal from reaching the cells in the dark area at all. (**Bottom panels**) The force of the strand, normalized to the amplitude of the isometric force of the simulated 1D strand at a stimulation frequency of 1 Hz before the ischemia onset. At *D* = 150 mm^2^/s the force (lower right panel) fluctuates slightly around 0.29 during every 20 twitches (see details in the text).

Comparison of Figure 1 and Figure 2 may initially suggest that, despite the permanent cell-to-cell interaction, both electrical and especially mechanical performance of the cardiomyocytes in the 1D tissue during the development of acute ischemia differ little from the corresponding performance of the single myocyte. However, for the 15 min phase, this initial impression turns out to be misleading. The APD and force patterns shown in Figure 2 are characteristic only of the first 18 stimuli after the onset of this phase; thereafter, they are disrupted dramatically. In response to the 19th stimulus in the 1D tissue model, we observed a block of both mechanical activity and electrical excitation in the myocardial tissue (Figure 4, left panels).

Table 1 shows the cardiac safety factor values for different stages of ischemia simulated in the 1D strand. As ischemia progresses, the safety factor diminishes, but remains above 1 until the block occurs (SF = 1.28 at the 5th minute of ischemia, and SF = 1.16 at the 10th minute of ischemia). Meanwhile, the occurrence of the block is accompanied by a decrease in SF to 0.85.

As a result of the conduction block, all cardiomyocytes in the 1D strand lost their ability to contract (Figure 4, left panels); that is, no active systolic force is generated in response to stimulation (Figure 4, lower-left panel). Moreover, even the residual spikes of the membrane potential of cardiomyocytes (Figure 4, upper-left panel), which were insufficient to trigger mechanical activity, gradually fade away as the residual excitation propagates along the first 20% of the strand length. In the remaining 80% of the strand, cells maintain a stable resting potential without any noticeable disturbances.

The 1D model revealed a possible compensatory mechanism that contributes to preventing conduction block in ischemic myocardium. Such a mechanism turned out to be a slowing down of the diffusion coefficient of the electrical signal along the strand during ischemia, which is associated with decreased conductance of intercellular gap junctions.

It should be emphasized that the decrease in the value *D* during the simulation of the 15 min ischemia (the ***IM*2_15_** model) further slowed down the excitation wave compared to the slowing caused solely by the decrease in *i_Na_*. Nonetheless, when the value *D* = 300 mm^2^/s used in the ***IM*1_15_** model was reduced by a factor of 1.5 (*D* = 200 mm^2^/s) in the ***IM*2_15_** model, the conduction velocity decreased to 0.6 m/s. Under these conditions, the conduction block became transient, occurring intermittently after every two or three regular contractions at a pacing rate of 1 Hz (Figure 4, middle panels).

Moreover, a twofold reduction in the electrodiffusion coefficient to *D* = 150 mm^2^/s in the ***IM*2_15_** model resulted in a further decrease in conduction velocity to 0.4 m/s and an elimination of the conduction block (Figure 4, right panels). The mechanical activity exhibited low-frequency oscillations, with beat-to-beat gradual increases in peak force (up to about 10%) occurring over the course of 10 twitches. This was followed by a subsequent decline over the next 10 twitches.

If the diffusion coefficient is reduced below *D* = 150 mm^2^/s in the ***IM*2_15_** model, complete conduction block recurs. At this critically low *D*, diffusion is too weak to support excitation propagation; combined with the reduced *i_Na_*, the tissue loses the ability to sustain both electrical and mechanical activation.

We suppose that even after fifteen minutes of ischemia, the uncoupling of gap junctions occurs gradually rather than instantaneously. In other words, gap junction conduction also decreases gradually, and its resulting level may vary. Accordingly, in our simulations, we found it appropriate to decrease the electrodiffusion coefficient *D* in a gradual way, starting from the initial value of *D* = 300 mm^2^/s, reflecting the normal gap junction conduction in the model. Notably, for 200 mm^2^/s < *D* < 300 mm^2^/s, the excitation block arose in the model in the same manner as at *D* = 300 mm^2^/s.

Our simulations highlight two factors that slow electrical wave propagation during ischemia, which have opposite effects. The first is the reduction in excitability caused by a decrease in *i_Na_* in cardiomyocytes, which promotes conduction block. The second is the slowing resulting from reduced gap junction conductance, which helps to prevent conduction block.

## 3. Discussion

Reduction in the conduction velocity of the excitation wave during the development of acute cardiac ischemia is a well-known phenomenon [21]. Closely related to this slowing of excitation is the occurrence of conduction block, which in turn can serve as a substrate for reentrant circuits within the walls of the heart chambers. One of the primary reasons for the slowing of the excitation wave is the reduction in the fast sodium current (*i_Na_*) in ischemic cardiomyocytes, which significantly reduces the rate of cellular depolarization and diminishes the electrotonic current. Furthermore, beginning at approximately 15 min of acute cardiac ischemia, partial closure of gap junctions begins to reduce intercellular conductance [11,22]. The contribution of these mechanisms to excitation slowing and the resulting conduction block in ischemic myocardium remains unclear. In particular, it has been supposed that “gap junction uncoupling may partially compensate for reduced Safety Factor (due to reduced *i_Na_* availability) preserving slow but still effective conduction” [9].

We have used mathematical modeling both to test this hypothesis and to elucidate the mechanisms underlying the occurrence of conduction block in ischemic myocardium. Previous studies have employed mathematical models to analyze factors contributing to conduction slowing and block, such as reduced *i_Na_* and gap junction uncoupling [5,6,7,8]. First, in these works, the factors were considered mainly individually, and partly in combination—but not in direct opposition to one another with respect to facilitating or hindering the block. Even when the factors were compared, the question was a different one: which mechanism of wave slowing contributes more to the block? That is, the question was not about the opposing directions of these factors in terms of their impact on the block, but rather about their co-direction, which differed in effectiveness. Secondly, while these studies involved mathematical modeling to explore the factors affecting the block, they did not focus on the underlying mechanisms.

Therefore, we sought to address this gap by focusing our attention on the mechanisms underlying the conduction block during acute ischemia.

The factors promoting the block were considered in the mentioned works not only under conditions of acute ischemia. For example, in the article by Campos et al. [8], the authors considered idealized 2D models of myocardial tissue containing a post-infarction scar. They computed statistics on the probability of conduction block in 2080 simulations with various combinations of parameters promoting the slowing of conduction, including *i_Na_* and *σ_m_* (the latter is the electrodiffusion coefficient representing in their work the tissue conductivity). Highly reduced *i_Na_* in their simulations resulted in the tissue becoming unexcitable. However, reductions in *σ_m_* alone were not sufficient to induce conduction block, while decreased *σ_m_* combined with reduced *i_Na_* could promote conduction block.

Shaw & Rudy used a 1D homogeneous model, composed of uniform cardiomyocytes coupled by resistive gap junctions, to investigate the influence of various cellular mechanisms on excitation propagation [5]. They employed two main criteria to assess propagation capability: conduction velocity and safety factor (SF), an index used to estimate the success of action potential propagation from excited to adjacent unexcited cells within cardiac tissue. According to Shaw and Rudy’s definition (Equation (12)), the SF of a single cardiomyocyte is calculated as the ratio of the charge generated by the cell during its own depolarization and by the depolarizing downstream cells to the charge the cell receives from the upstream tissue [5]. Therefore, if the SF > 1, excitation successfully propagates downstream, indicating that the charge generated by the cell during excitation exceeds the charge required to depolarize neighboring cells.

Shaw and Rudy [5] demonstrated that in their electrophysiological 1D model, a reduction in the maximal conductance of *i_Na_*, which determines membrane excitability, produces a monotonic decrease in both conduction velocity and the SF, resulting in conduction block when maximal *i_Na_* conductance is reduced to 10% of its initial value. Similar results concerning the significant effect of *i_Na_* variability on SF were independently reported by Boyle et al., who employed their own formulation of this characteristic [23].

Importantly, a gradual uncoupling of cells by a decrease in gap junction conductance led, in the simulations presented by Shaw and Rudy [5], to a monotonic decrease in conduction velocity accompanied by an increase in the SF. Maximum SF values occurred when intercellular coupling was reduced by a factor of about 15 compared to the normal value. This trend of increasing SF reversed only at very low values of gap junction conductance. Conduction block occurred only when the conductance was reduced by nearly 600-fold, that is, essentially due to the electrical uncoupling of the cardiomyocytes. In other words, within the broad physiological range of the gap junction conductance, a decrease in this conductance caused an increase in the SF. These results indicate that slowing of excitation conduction caused either by reduced membrane excitability or by uncoupling of the cells can have opposite effects on the SF. However, the authors did not analyze how a simultaneous decrease in both *i_Na_* and electrical interaction between the cells may affect the SF.

In our model, we simulate and evaluate the simultaneous contribution of both factors under particular conditions of acute ischemia. Moreover, in our simulations (unlike those presented by Shaw and Rudy [5]), *i_Na_* decreases automatically in response to hyperkalemia, rather than due to a parametric decrease in its conductance. This seems to be an important circumstance. Acidosis decreases the conductance of *i_Na_* during ischemia, but it cannot reduce this current by an order of magnitude. Such an order-of-magnitude reduction is a necessary prerequisite for excitation block, both in the Shaw–Rudy 1D model and in ours. Indeed, pH during ischemia can decrease by no more than 1 unit from its normal level (from ~7.2 to ~6.2) [24], and this decrease reduces sodium conductance by approximately 25%. In other words, a 90% decrease in *i_Na_* conductance does not occur under ischemic conditions. However, according to our simulations, a multiple drop in the *i_Na_* current still occurs under these conditions without any modification of its conductance parameter and this still leads to excitation block. Specifically, we have shown that such an ischemic factor as hyperkalemia by itself does cause this multiple drop in the *i_Na_* current. At first glance, this finding of ours might seem counterintuitive; however, the analysis we presented in Figure 3 in the Section 2 reveals the mechanism underlying the effect of hyperkalemia on the *i_Na_* current drop.

It should be noted that in another study, Shaw and Rudy examined in detail the factors responsible for excitation wave slowing down specific to ischemia in their 1D model [6]. However, in that work, firstly, they did not use SF estimates, and secondly, they limited their investigation to the stages of ischemia before the onset of gap junction uncoupling. In other words, the contribution of this uncoupling to wave slowing and to the emergence or disappearance of the excitation block was not addressed in that article at all. Consistent with their earlier findings [5], Shaw & Rudy [6] showed that the acidotic decrease in *g_Na_* led to conduction block only at a 90% reduction—a level well outside the range typical of ischemia. Only hyperkalemia, considered both alone and in combination with other factors within the ischemia simulations of Shaw and Rudy [6], led to excitation block. This finding echoes our results.

Another important difference between our data and the results of Shaw and Rudy [6] is that, even when all ischemic factors were taken into account in their work, the block occurred only at high ischemic levels of the extracellular potassium ([K^+^]*_o_* = 11 mM). In contrast, in our model the block occurred at a significantly lower [K^+^]*_o_* = 9.4 mM, even without taking acidosis into account. Moreover, anoxia in our 15 min ischemia model producing the block is less pronounced than in that of Shaw and Rudy: [*ATP*]*_i_* = 4.5 mM (in our model) vs. [*ATP*]*_i_* = 3 mM (in Shaw and Rudy work [6]). Unlike the purely electrophysiological models used in previous studies [5,6], our model incorporates both intracellular and intercellular electromechanical coupling in a multicellular myocardial sample. This fundamental difference, as we demonstrate below, highlights the significant role of not only electrical but also mechanical interactions between cardiomyocytes in the mechanisms underlying the block.

*The role of mechano-electric and mechano-calcium feedback in conduction block during ischemia*

Let us discuss how exactly this feedback in cardiomyocytes, along with mechanical interaction between cells in the 1D strand (***IM*1_15_** model), initiates the block under the parameters of the 15 min ischemia specified in our model.

As explained below, the block arises due to several factors related to the electromechanical interaction of cardiomyocytes in the tissue, which, from stimulus to stimulus, additionally reduce the voltage-dependent activity of Na^+^ channel gates.

**Figure 5:**
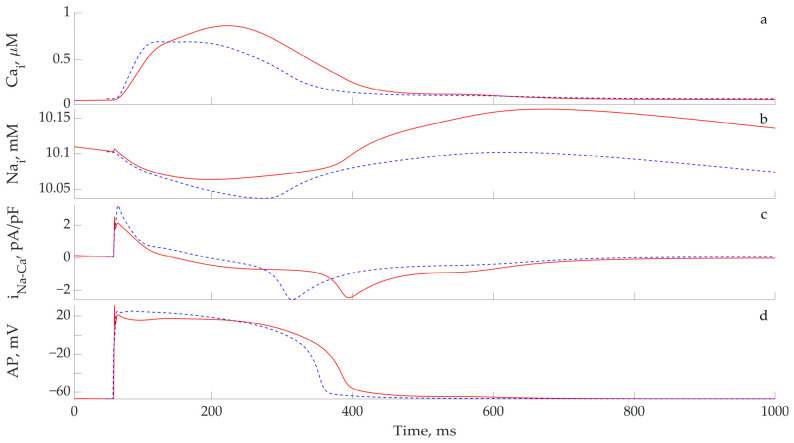
Comparison of Ca^2+^ transients (**a**), intracellular Na^+^ concentration dynamics (**b**), Na^+^-Ca^2+^ exchange current (**c**), and action potential (**d**) in the first cell of the 1D ***IM*1_15_** model during the last twitch before the onset of the block (solid lines), and in a specially tuned model of the single isolated cell with identical parameters (dashed lines), which is stimulated with the same initial conditions as this cell in the 1D strand (see the text for more details). Time on the axes of the panels is shown starting from the moment of the last stimulus before the onset of the block in the ***IM*1_15_** model.

Right in the first cell of the ***IM*1_15_** model, the *i_Na_* current becomes even smaller than in an isolated cardiomyocyte with the same ischemia-induced disorders, and gradually decreases from stimulus to stimulus during the first 18 twitches after the start of the 15 min ischemia simulation in the strand. This additional decrease gradually brings the *i_Na_* current in this cell close to its non-excitation threshold. To analyze the reason why the first cell of the ***IM*1_15_** model crosses this threshold, let us consider in more detail its behavior after the last twitch before conduction block (Figure 5, solid lines). To investigate this, we compare this behavior with a specially tuned model of the same cell in isolation, which is stimulated with the same initial conditions as this cell in the 1D strand; however, unlike its copy in the strand, it does not communicate electrically and mechanically with the neighbors (Figure 5, dashed lines).

(1) An increase in the Ca^2+^ transient in the first cell of the 1D tissue model compared to isolation (Figure 5, upper panel) is caused by the mechano-calcium feedback. Indeed, shortening of the first cell occurs immediately after its activation unlike in isolation, since this shortening is resisted only by the initially passive (not yet activated), i.e., mechanically weak part of the ***IM*1_15_** model. This shortening, in turn, causes an additional increase in the Ca^2+^ transient due to the cooperative effect of the mechano-dependent cross-bridges on the kinetics of CaTnC complexes (see the description of our cardiomyocyte mathematical model in the Section 4 below).
(2) Due to this increase in the Ca^2+^ transient, the Na^+^-Ca^2+^ exchanger (NCX) removes Ca^2+^ faster due to the concentration gradient. Consequently, Na^+^ enters the tissue cell faster than the isolated cell. Therefore, at the moment of the next stimulus, the Na^+^ concentration in the first cell of the 1D ***IM*1_15_** model is higher than during the previous stimulus applied to this cell, whereas this does not occur in the tuned model of the isolated single cell (compare the solid and dotted lines in the bottom panels of Figure 5). This higher Na^+^ concentration reduces the electrochemical gradient across the cell membrane, causing the *i_Na_* current to be smaller at the time of the next stimulus compared to the previous one. This current was already slightly above its threshold, which is sufficient to excite the cell in response to the previous stimulus, and, consequently, now it falls below this threshold. Thus, an excitation block occurs in the ***IM*1_15_** model.

It is noteworthy that, according to Figure 5, not only mechanical but also electrical interaction of the examined cell with its surroundings in the model ***IM*1_15_** contributes to the increase in intracellular Na^+^ concentration and thus to the decrease in the *i_Na_* current upon the next stimulus. However, this electrical contribution is limited to the first 200 ms and is quantitatively much smaller than the ultimate contribution of the mechanical interaction at the time of the subsequent stimulus. This effect arises because electrotonic current drain from the cell within the tissue reduces its AP amplitude compared to the isolated cell during the first 200 ms (Figure 5d). This interval coincides with the reverse mode of the NCX. The lower AP amplitude results in a smaller NCX current in the reverse mode in this cell within the model ***IM*1_15_** than in the isolated cell (Figure 5c). Consequently, less Ca^2+^ is brought in by this exchanger and, correspondingly, less Na^+^ is removed from the cell during this initial 200 ms period in the model ***IM*1_15_** cell compared to the isolated cell (Figure 5a,b). In other words, slightly more Na^+^ remains in the cell primarily due to its electrical interaction with other cardiomyocytes in the model ***IM*1_15_** during the first 200 ms.

However, by 200 ms, the reverse mode of the NCX ends. Subsequently, the higher Na^+^ concentration in the strand cell compared to the isolated cell is maintained and increased by the forward mode of the NCX. This late accumulation is driven by the higher cytosolic Ca^2+^ level in the strand cell. As established above, that higher Ca^2+^ level arises from two sources: the mechanical interaction with other strand cells, which produces prominent shortening of the cell, and the mechano-calcium feedback within it.

A similar comparison between the first cell of the 1D ***IM*1_15_** model and 18 individual models of the same cell in isolation, each independently tuned to its specific initial conditions corresponding to one of the first 18 stimuli, helps clarify why *i_Na_* in the first cell of the 1D ***IM*1_15_** model is lower compared to an isolated cardiomyocyte specifically tuned to that particular stimulus, as well as why it progressively decreases with subsequent stimulations.

**Figure 6:**
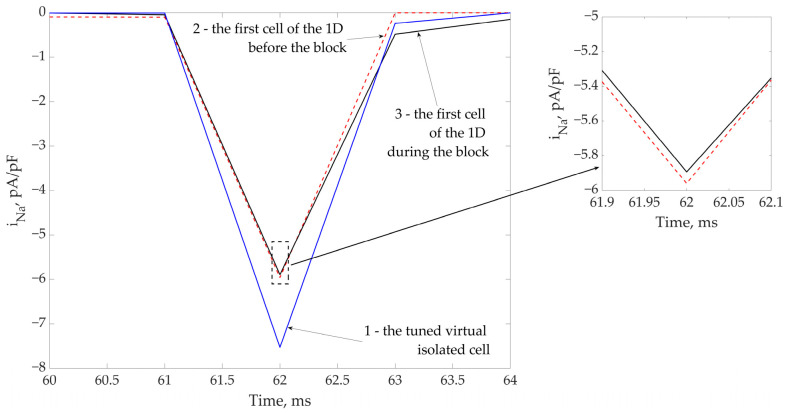
Fast sodium current *i_Na_* in the tuned virtual isolated cell (*trace* 1) and in the first cell of the 1D ***IM*1_15_** model immediately before the block (*trace* 2) and during the block onset (*trace* 3).

Our analysis reveals that the mechanical interaction of ischemic cardiomyocytes (specifically, the redistribution of their lengths during mechanical twitches) is a key factor. Through intracellular mechano-calcium feedback, this interaction gradually reduces *i_Na_* in these myocytes from one stimulus to the next, ultimately blocking their excitation in the 1D ***IM*1_15_** model (Figure 6).

To finally verify this, we performed the following numerical experiment. In the ***IM*1_15_** model, we “froze” the length of the strand itself and the lengths of all its cardiomyocytes at the level determined by the applied preload, while keeping all other parameters and initial values the same as before. In this way, we excluded the dynamic redistribution of cardiomyocyte lengths following strand stimulation, i.e., we eliminated the dynamically changing influence of mechanics on Ca^2+^ kinetics and on electrical activity via intracellular mechano-calcium feedback. In fact, in this numerical experiment, we simulated 15 min ischemia in a purely *electrophysiological* 1D model of human myocardium. In the same ischemic conditions (in particular, at the same level of hyperkalemia as in the electromechanical model, [K^+^]*_o_* = 9.4 mM), the block did not occur in this electrophysiological model. A block was only achieved by increasing [K^+^]*_o_* approximately 1.5-fold (i.e., to 14.5 mM) in this model with eliminated mechanical interaction between cardiomyocytes. This result demonstrates that seemingly minor mechano-dependent changes in *i_Na_* are in fact critical for the initiation of conduction block. In the absence of mechano-electric feedback in the strand, block does not occur at the same [K^+^]*_o_*, but only at a substantially higher level.

*Conduction block in a case of local ischemia*

**Figure 7:**
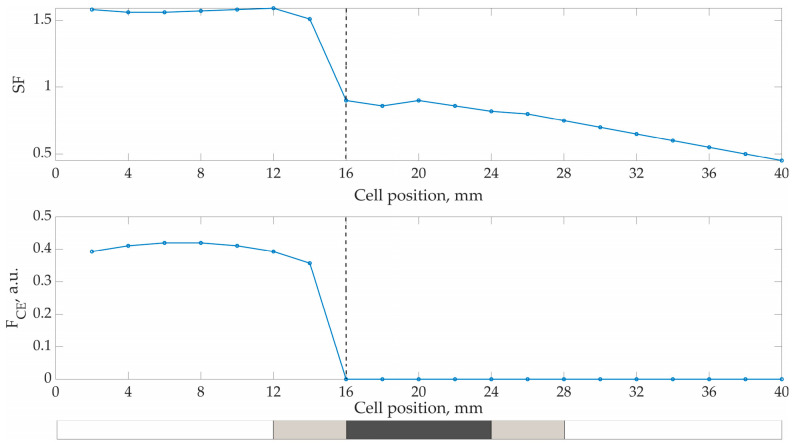
Safety factor (SF, upper panel) and contractile element force (F_CE_, bottom panel) in cardiomyocytes of a 1D strand during local ischemia at 15 min. The traces show the response to the 36th stimulus applied to the left edge of the strand (pacing rate 1 Hz). The bar under the panels indicates the strand regions: normal areas (white), border zone (light gray), and ischemic area (black). All parameters in the border zone change linearly from normal to ischemic values. Symbols on the curves mark the cells for which SF and F_CE_ values were measured (values normalized to the strand force in normal conditions). The vertical dotted line marks the cell where the conduction block occurs.

The simulations discussed above focused on a block of excitation in uniformly ischemic 1D myocardial tissue, similar to the simulations presented by Shaw and Rudy [6]. We employed a uniformly ischemic 1D strand to simplify the analysis of mechanisms underlying the block. However, in the real heart, an ischemic region is typically surrounded by healthy myocardium and interacts with it both electrically and mechanically. Therefore, we additionally simulated a 1D strand where the ischemic segment comprises only 10% of the total length, with the border zone occupying 5% of the length on each side of the ischemic zone (as indicated by the bar under the panels in Figure 7). For simplicity, all cell parameters in the border zone vary linearly from the normal to the ischemic segment. The excitation wave propagated from left to right along the strand. Under 15 min local ischemia conditions, a propagation block occurred in the leftmost cell of the ischemic segment in response to the 36th stimulus. Specifically, in this cell, the SF curve drops below the excitability threshold level of 1 (SF = 0.9; Figure 7, upper panel), and the force of the contractile element F_CE_ (i.e., active force of the cell) fell to zero (Figure 7, lower panel). In other words, both electrical and mechanical activity ceased in this cell. In all the subsequent cells along the strand (from left to right Figure 7) SF continued to decrease as low as 0.5. Mechanical activity was also absent in these cells.

Note that under local ischemia conditions, the block generally does not necessarily occur exactly at the border of the ischemic area, as was the case in Figure 7. For example, increasing the width of the border zone by a factor of two compared to Figure 7 shifts the block location 1 mm into the ischemic area.

Finally, in both our local and global models of the 15 min ischemia, a moderate decrease in gap junction conductance promoted the overcoming of the conduction block. In continuous models of myocardial tissue, a decrease in gap junction conductance is usually represented by a decrease in the electrodiffusion coefficient *D* (see, e.g., the aforementioned study by Campos et al. [8]). We also implemented this method. As shown in Figure 4, when *D* was reduced by a factor of 1.5 in the entirely ischemic strand (*D* = 200 mm^2^/s), the block was overcome in response to two out of three consecutive stimuli applied at a pacing rate of 1 Hz. When *D* was reduced twofold (*D* = 150 mm^2^/s), the block was completely eliminated. We obtained an entirely similar result when simulating local 15 min ischemia in the 1D tissue model.

Thus, our modeling results confirm the hypothesis put forward by Dhein [9], suggesting that a slower efflux of charge from the cell can compensate for the reduced *i_Na_* current in ischemic cardiomyocytes, thereby maintaining their electrical excitability. However, our simulations also revealed that this compensation is incomplete from the perspective of the mechanical activity of the myocardium. Specifically, the force amplitude of the virtual preparation not only decreased several-fold compared to the healthy sample, but also exhibited unstable behavior, with periodic slight increases and decreases from one twitch to the next. Moreover, a further reduction in the diffusion coefficient below *D* = 150 mm^2^/s restored the conduction block.

Of course, a further increase in hyperkalemia above [K^+^]*_o_* = 11.5 mM leads to the block even for *D* = 150 mm^2^/s.

In other words, our simulations predict that under acute ischemia, a compensatory range for reduced intercellular electrical communication due to the closure of gap junctions does exist, but it is not very wide.

## 4. Materials and Methods

### 4.1. Cellular Simulations

We simulated excitation–contraction coupling in the human cardiomyocyte using the electromechanical model (*TP+M* model) [25]. This model integrates the electrophysiological *ten Tusscher*–*Panfilov* model of the human ventricular cardiomyocyte [26] with our description of mechanical behavior adapted from the *Ekaterinburg*–*Oxford* (*EO*) electromechanical model. The electrical and mechanical components of the *TP+M* model are coupled through a description of intracellular calcium dynamics, which plays a role in both action potential (AP) generation and the generation of myocardial force and shortening [10]. Equations of the *TP+M* model have been described in detail previously [25] and were subsequently slightly modified. The key features of these equations are summarized below, along with a brief description of the 1D tissue model, which incorporates models of the cardiomyocytes that form this tissue.

### 4.2. 1D Simulations

Our study employed a sample of human myocardium represented as a continuous 1D mathematical model of mechanically and electrically coupled cardiomyocytes connected in series. This approach is based on a previously published model of the 1D virtual strand [16,27], in which the original *EO* model of a guinea pig cardiomyocyte was replaced with the electromechanical model *TP+M* of a human cardiomyocyte outlined above.

This section provides a brief overview of the key features of the *TP+M* model (microlevel) and the 1D virtual strand (macrolevel). For comprehensive details on the model development, please refer to the respective articles.

**Figure 8:**
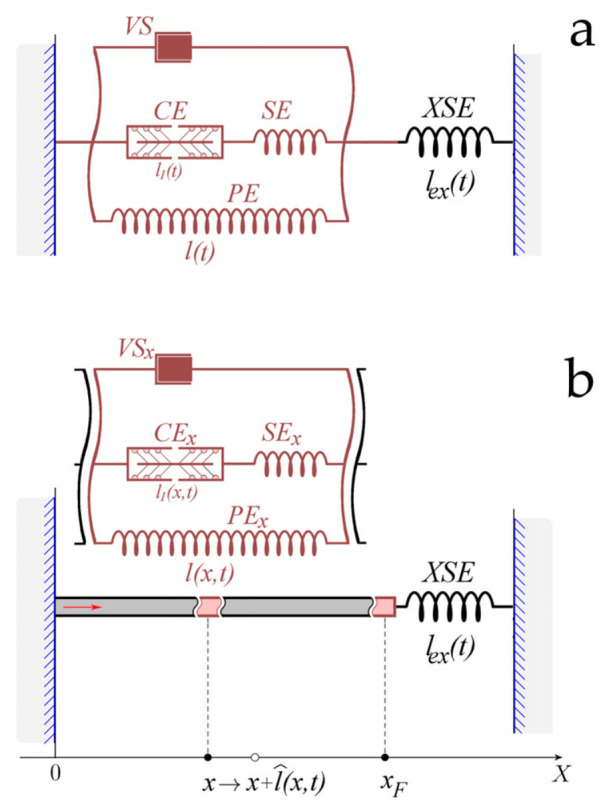
Schematic representation of (**a**) single-cell mechanics and (**b**) mechanics of a 1D virtual strand of cardiac tissue under isometric conditions. The inset in panel (**b**) shows the rheological scheme of a single cardiomyocyte (cell *x*) used at point *x* of the strand. (**a**,**b**): The contractile element *CE* (*CE_x_*) is connected in series and parallel to passive elastic elements (*SE*, *SE_x_* and *PE*, *PE_x_*, respectively). A viscous element *VS* (*VS_x_*) is placed in parallel with the parallel elastic element *PE* (*PE_x_*). *XSE* is an external in-series elastic element (relative to the cells). Current changes in the lengths of *PE*, *CE* (*PE_x_*, *CE_x_*) and *XSE* normalized to their slack lengths are denoted as *l*(*t*), *l*_1_(*t*) (*l*(*x*,*t*), *l*_1_(*x*,*t*)) and *l_ex_*(*t*), respectively. (**b**): The distance [0, *x_F_*] corresponds to the slack length of the virtual 1D strand. The variable $\hat{l}(x,t)$ represents the displacement of cell *x* from its initial position in the unstretched and relaxed strand. The excitation wave propagates from the left to the right end of the 1D strand (as indicated by the arrow).

The 1D virtual tissue strand is represented as a static continuous medium. Each point is identified by the Lagrangian coordinate *x* representing a specific cardiomyocyte within the strand at the macrolevel. By associating the left end of the strand with *x* = 0, the slack length of the strand is defined by the coordinate of its right end, *x* = *x_F_* (Figure 8). In the rheological scheme of the 1D model, an external serial elastic element (*XSE*) represents the compliance at the cut muscle edge, which would be connected to a servomotor arm in experiments on multicellular muscle strips [28].

The mechanical behavior of each cardiomyocyte *x* during the contraction–relaxation cycle depends on both its intrinsic contractile activity and the dynamic mechanical environment within the 1D virtual strand. This environment is determined by the contraction of all other cardiomyocytes within the strand. The rheological scheme of a single cardiomyocyte *x* (Figure 8) includes a contractile element (*CE_x_*), representing the sarcomeres, and passive elastic (*PE_x_*, *SE_x_*) and viscous (*VS_x_*) elements. These passive elements correspond to passive mechanical properties of the myocardium but also contribute to the overall mechanical behavior of the active cell.

The variable *l*(*x*, *t*) represents the relative length change in cell *x* per sarcomere, normalized to its slack length. When the strand is stretched or shortened, cell *x* shifts from its reference position in the resting strand by a distance denoted as $\hat{l}(x,t).$ Thus, the current time-dependent position of cell *x* during the contractile cycle is $x+\hat{l}(x,t)$ (Figure 8). Consequently, the total extension of the 1D strand length relative to its slack length is $l_{m}(t)=\hat{l}(x_{F},t)+l_{ex}(t),$ where $l_{ex}(t)$ is the elongation of the external serial elastic element (*XSE*), normalized to the slack length of the strand.

During isometric contraction, the strand length remains constant: $l_{m}(t)=l_{m}(0)\;\equiv \;const,$ as determined by the preload (ρ) applied to the strand before the contraction cycle. The length of *XSE*, $l_{ex}(t),$ under isometric conditions, is determined by the initial prestretch $l_{ex}(0)$ and the cumulative deformation of all cells of the strand.

We postulate that the local instantaneous deformation of the strand at any point *x* and time *t* at the macroscopic level (i.e., $\frac{\partial \hat{l}(x,t)}{\partial x}$) equals the current length change of the corresponding cardiomyocyte *x* at the microscopic level:

(1) $$\frac{\partial \hat{l}(x,t)}{\partial x}=l(x,t).$$

The boundary conditions for Equation (1) at *x* = 0 and *x* = *x_F_* during isometric contractions are

(2) $$\hat{l}(0,t)=0,$$

(3) $$\hat{l}(x_{F},t)+l_{ex}(t)=l_{m}(0).$$

At the microscopic level, the length change *l*(*x*,*t*) of each cell *x* is initiated by the contraction of its contractile element (*CE_x_*). The underlying processes, including calcium activation kinetics and force generation of the actin–myosin cross-bridges in sarcomeres, are formalized in the equations of the *TP+M* cellular submodel. A brief description of these processes is provided below.

The dynamics of the membrane potential and ionic currents in each cell *x* are described by the electrophysiological component of the *TP+M* model. The macroscopic electrophysiology of the 1D strand is governed by the depolarization wave that propagates from *x* = 0 to *x* = *x_F_*. The conduction velocity of this wave depends on both the tissue conductance at the macroscopic level and the AP development in individual cardiomyocytes, and their electrotonic interaction, at the microscopic level.

**Figure 9:**
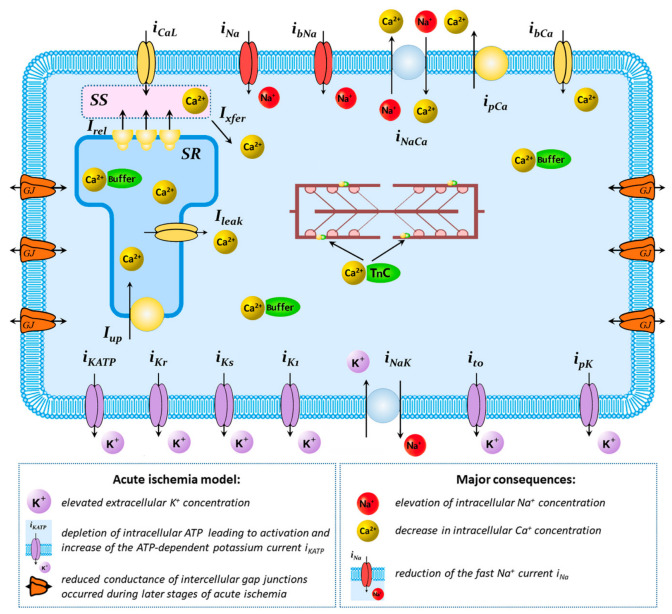
Schematic representation of ionic membrane currents and intracellular Ca^2+^ handling in the single cardiomyocyte model (*TP+M* model). Ion translocation across the sarcolemma involves voltage- and time-dependent currents: L-type Ca^2+^ current (*i_CaL_*), background Ca^2+^ current (*i_bCa_*), fast Na^+^ current (*i_Na_*), background Na^+^ current (*i_bNa_*), inward rectifier K^+^ current (*i_K_*_1_), transient outward current (*i_to_*), rapid and slow delayed rectifier K^+^ currents (*i_Kr_*, *i_Ks_*), and plateau K^+^ current (*i_pK_*). The model also includes the specific ATP-sensitive K^+^ current (*i_KATP_*) to simulate ischemic conditions. Additional modeled currents include the sarcolemmal Ca^2+^ pump (*i_pCa_*), Na^+^-K^+^ pump (*i_NaK_*), and Na^+^-Ca^2+^ exchanger (*i_NaCa_*). In the 1D tissue model, the electrical propagation between cardiomyocytes is mediated by non-selective ionic currents across gap junctions (GJs). Specific ionic currents through GJs are not explicitly included in either the isolated single cardiomyocyte model or the cellular submodels of the continuous 1D model. The GJ conductance in the 1D model is represented by the electrodiffusion coefficient (Equation (4)). Intracellular Ca^2+^ dynamics comprise release from the sarcoplasmic reticulum (*SR*) via ryanodine receptors into the subspace (*SS*) (*I_rel_*), diffusion between the SS and bulk cytosol (*I_xfer_*), active uptake into the SR (*I_up_*), where Ca^2+^ is partially buffered (*Buffer* in SR), and a passive SR leak (*I_leak_*). Cytosolic Ca^2+^ is buffered by ligands (*Buffer* in cytosol) and binds to troponin C (*TnC*) to initiate sarcomere contraction. The bottom-left box specifies the key conditions of acute ischemia simulated in the single cardiomyocyte and 1D models in this study. The bottom-right box outlines the major subsequent ionic alterations responsible for ischemia-induced changes in action potential morphology, force generation, and conduction block. These consequences are examined in detail in the Section 2 and Section 3.

Figure 9 illustrates the scheme of electrophysiological processes and intracellular Ca^2+^ handling in the *TP+M* model. The model incorporates the major sarcolemmal sodium, potassium, and calcium currents, as well as Ca^2+^ translocation processes, responsible for AP generation and resting potential maintenance. For this study, we added the ATP-sensitive K^+^ outward current (*i_KATP_*), which is activated under ATP depletion conditions, to simulate ischemia (see Section 4.3 for details).

Electromechanical coupling in the cardiomyocyte model is mediated by intracellular Ca^2+^ kinetics. The following sequence of events is implemented. The AP, initiated in the cardiomyocyte by *i_Na_*, induces Ca^2+^ influx through the L-type Ca^2+^ channels (*i_CaL_*). This influx triggers Ca^2+^-induced Ca^2+^ release from the sarcoplasmic reticulum (SR). The resulting increase in cytosolic Ca^2+^ promotes its binding to the regulatory protein troponin C, forming CaTnC complexes. This binding enables cross-bridge (XB) formation, leading to sarcomere force generation and sliding of the actin filaments relative to the myosin filament, which shortens the sarcomere. Cardiomyocyte relaxation following AP repolarization is associated with Ca^2+^ dissociation from troponin C and its removal from the cytosol via extrusion from the cell and reuptake into the SR.

The cardiomyocyte mathematical model incorporates not only intracellular mechanisms of electromechanical coupling but also a pathway of mechano-electrical feedback known as calcium–myofilament interactions [29]. This pathway is based on the mechano-dependence of Ca^2+^ handling and stems from the mechano-sensitivity of the troponin C (TnC) affinity for Ca^2+^. This mechano-sensitivity, in turn, is governed by the cooperative kinetics between mechano-dependent cross-bridges and Ca^2+^-troponin C (CaTnC) complexes [28,30]. Specifically, this cooperative kinetics promotes an increase in the intracellular Ca^2+^ concentration ([Ca^2+^]*_i_*) in response to the cardiomyocyte shortening (*ibidem*). Thus, mechano-induced alterations in Ca^2+^ kinetics modify the Ca^2+^ transient, thereby influencing AP generation and providing mechano-electrical feedback.

To simplify the description of excitation propagation along the 1D virtual strand, we neglect relatively small dynamic changes in the instantaneous wave propagation path. These changes arise from moderate compression or stretching of the strand’s segments during the isometric contraction–relaxation cycle. In other words, the equation is formulated in Lagrangian coordinates:

(4) $$\frac{\partial V(x,t)}{\partial t}=D\cdot \frac{\partial^{2}V(x,t)}{\partial x^{2}}-\frac{1}{C_{m}(x)}\cdot \sum i_{ion}(x,t),$$

where *C_m_*(*x*) is the membrane capacitance of cell *x*, $\sum i_{ion}(x,t)$ represents all local transmembrane ionic currents in the cell *x* (as shown in Figure 9), and *D* is the electrodiffusion coefficient. In continuous myocardial medium, this coefficient reflects the conductive properties of gap junctions between cardiomyocytes and determines the conduction velocity. Equation (4) describes the coupling between microscopic events (AP generation due to changes in membrane ionic currents in cell *x*, represented by the second term) and macroscopic processes (excitation wave propagation along the 1D strand, represented by the first term).

The equations describing all the ion currents are standard. Specific formulations for these currents can be found elsewhere [25,26]. Here we present only the equation for the fast sodium current *i_Na_*, as its components are essential for analyzing the mechanisms underlying the phenomena modeled in this work:

(5) $$i_{Na}(x,t)=g_{Na}\cdot m^{3}\cdot h\cdot j\cdot (V(x,t)-E_{Na}),$$

where *g_Na_* is the maximal *i_Na_* conductance; *m* is the voltage-dependent open probability of the activation gate; *h* and j are the voltage-dependent open probabilities of the fast and slow inactivation gates (see differential equations for *m*, *h* and *j*, in ten Tusscher and Panfilov [26]); and *E_Na_* is the sodium reversal potential.

The boundary conditions for Equation (4) are defined as follows:

(1) A brief stimulating depolarizing current *i_stim_*(*t*) = −52 pA/pF is applied to the left end of the strand (at point *x* = 0) for 1 ms to initiate excitation in the boundary cell:

(6) $$D\frac{\partial V(0,t)}{\partial x}=-\frac{1}{C_{m}}i_{stim}(t).$$

No additional stimulating currents are applied to other cells in the strand; their depolarization results from the propagation of an excitation wave.

(2) The right end of the strand (*x* = *x_F_*) is electrically isolated, meaning no current flows through this boundary point:

(7) $$\frac{\partial V(x_{F},t)}{\partial x}=0.$$

The initial conditions are set as follows. The resting potential value at *t* = 0, which is identical to that in the isolated cardiomyocyte *TP+M* model, is applied to all cells in the strand:

(8) $$V(x,0)=V_{rest}(x).$$

The initial values of all other state variables in the cardiomyocyte *TP+M* models used in the 1D virtual strand are the same as in the single cardiomyocyte presented by the *TP+M* model, unless specifically stated otherwise in the numerical experiment protocol.

The full set of equations and parameters is provided in the Supplementary File.

The cardiomyocyte and 1D strand models were implemented in Python 3.12 (Python Software Foundation) with NumPy 1.26.4 and Numba 0.65.1 for JIT acceleration; the ODE systems were integrated with the CVODE solver (SUNDIALS 7.7.0) through the Assimulo 3.8.0 interface.

### 4.3. Simulation of Acute Ischemia

In this study, we simulated and compared the activity of the single cardiomyocyte and the 1D muscle strand during the first 15 min of acute ischemia: hyperkalemia (elevated extracellular K^+^ concentration, [K^+^]*_o_*) and anoxia due to the reduction in coronary blood flow. We first simulated the isolated cell under these conditions and then used these ischemic cardiomyocyte models to construct the 1D virtual strand, thereby investigating the effects of ischemia on the electrical conduction and mechanical function in the tissue.

To simulate hyperkalemia, the parameter representing extracellular K^+^ concentration ([K^+^]*_o_*) in the *TP+M* model was increased from a control value of 5.4 mM to 6.2, 8.0, and 9.4 mM, corresponding to 5, 10, and 15 min of acute ischemia, respectively. The value of [K^+^]*_o_* = 5.4 mM for the 0 min time point (simulating baseline healthy conditions) was adopted from the *ten Tusscher–Panfilov* model [26], which is integrated into the *TP+M* model as its electrophysiological component. This baseline value is itself based on experimental data from human ventricular cardiomyocytes [31,32].

Due to the lack of reliable, quantitative data on extracellular K^+^ concentration in the human myocardium during ischemia, the [K^+^]*_o_* levels for 5, 10, and 15 min of ischemia were adapted from the study by Shaw & Rudy [11], whose model parameters were primarily derived from the guinea pig data.

Preliminary experiments with the single *TP+M* model demonstrated that elevated [K^+^]*_o_* accurately mimics the experimentally observed depolarization of resting potential under ischemic conditions. However, the effect on AP duration was more complex: depending on other model parameters, increased [K^+^]*_o_* could lead to either AP shortening or prolongation. A similar finding was reported by Weiss et al. [33] in their analysis of extracellular K^+^ elevation in the *ten Tusscher–Panfilov* model.

Our analysis indicates that these divergent effects stem from the specific mathematical formulations of the inward rectifier K^+^ current (*i_K_*_1_) and the rapid delayed rectifier K^+^ current (*i_Kr_*), especially the former. The *i_K_*_1_ formulation is derived from the guinea pig *Luo–Rudy* cardiomyocyte model [34] and incorporates two opposing mechanisms influenced by [K^+^]*_o_*. One factor is the electrochemical gradient of K^+^ across the membrane, which tends to prolong AP as [K^+^]*_o_* rises. Conversely, the conductance of the *i_K_*_1_ channels increases monotonically with [K^+^]*_o_*, promoting AP shortening. Which trend ultimately dominates depends on the complex interplay of all ionic currents and feedbacks during the AP and is also influenced by the mechanical conditions of myocyte contraction.

The dependence of the maximal *i_K_*_1_ conductance (*g_K_*_1_) on [K^+^]*_o_* was established in experiments on various animal species [35,36,37], including the guinea pig [38], over a wide range of [K^+^]*_o_*, including mostly non-physiological values. This dependence was first formalized in a guinea pig cardiomyocyte model by Di Francesco and Noble [39]. Notably, both the *Luo–Rudy*, whose formulation of this dependence is used in the present *ten Tusscher*–*Panfilov* model, and *Noble–Di Francesco* models approximated the same experimental *i_K_*_1_ current–voltage data for different levels of [K^+^]*_o_* [38]. However, in their models, this dependence is implemented in completely different ways, indicating that both approximations are rough.

To correctly reproduce the combined effect of hypoxia and hyperkalemia in the *TP+M* model, specifically, AP shortening and reduced force generation, the analytical dependence of the conductance of K^+^ currents on [K^+^]*_o_* was modified. The exponent *n* of the ${\left(\frac{[K^{+}{]}_{o}}{[K^{+}{]}_{o,norm}}\right)}^{n}$ ratio was increased from 0.5 to 0.92 in the formulations of the conductances of *i_K_*_1_ and *i_Kr_* to facilitate the effect of hyperkalemia.

To simulate the effects of anoxia, which depletes intracellular ATP, we incorporated the ATP-dependent potassium current *i_KATP_* into the single cardiomyocyte *TP+M* model, using the formulation by Shaw and Rudy [11]. This formulation determines the conductance of this current as a function of the cytosolic concentration of ATP ([*ATP*]*_i_*) and [K^+^]:

(9) $$i_{KATP}(t)=g_{KATP}\cdot P_{ATP}\cdot {\left(\frac{[K^{+}{]}_{o}}{[K^{+}{]}_{o,norm}}\right)}^{n}\cdot (V(t)-E_{K}),$$

where for the cell at position *x* in the 1D strand, $g_{KATP}$ = 3.9 µS is the maximal channel conductance; [K^+^]*_o_*_,*norm*_ = 5.4 mM is the physiological extracellular K^+^ concentration; *n* = 0.24 is the sensitivity coefficient of *i_KATP_* conductance to changes in [K^+^]*_o_*; and E_K_ is the potassium reversal potential. The fraction of open ATP-dependent K^+^ channels ($P_{ATP}$), is given by a Hill-type expression that describes its nonlinear dependence on [*ATP*]*_i_*:

(10) $$P_{ATP}=\frac{1}{1+{\left(\frac{[ATP{]}_{i}}{k_{0.5}}\right)}^{h}},$$

where *k*_0.5_ represents the cytosolic ATP concentration for half-maximal channel activation (see below for its specific values under different conditions); *h* = 2.2 is the Hill coefficient, characterizing the slope of the dependence of the open channels on [*ATP*]*_i_*.

The half-maximal activation constant *k*_0.5_ of the Hill-type equation for the $P_{ATP}$ was modified from its original value in the Shaw and Rudy formulation [11] to improve the model’s physiological accuracy. In their model, *i_KATP_* is non-zero at physiological intracellular ATP concentrations, despite experimental evidence showing that intracellular ATP almost completely inhibits this current under normal conditions [40]. Following the approach of Abbasi and Clayton [41], we set *k*_0.5_ to increase progressively with the severity of anoxia, corresponding to decreasing [*ATP*]*_i_*. Thus, to simulate anoxia at 0, 5, 10, and 15 min of acute ischemia, the following pairs of [*ATP*]*_i_* and *k*_0.5_ were used for *i_KATP_* simulation: (6.8 mM, 0.042 mM), (6.0 mM, 0.117 mM), (5 mM, 0.212 mM), and (4.5 mM, 0.259 mM).

All modifications described above pertain to acute ischemic changes implemented in the cardiomyocyte models, representing the microlevel of the virtual 1D sample.

### 4.4. 1D Simulation of the Acute Ischemia

At the macrolevel of the myocardial sample, acute ischemia manifests as a slowdown in excitation propagation, which is confirmed by experiments on whole pig hearts [42] and on canine models [43]. This slowing results from several mechanisms, one of which is a consequence of intracellular ischemic alterations that directly impair electrotonic interaction between the cardiomyocytes. As demonstrated in the Section 2, our 1D model accurately reproduces this electrotonic response resulting from the ischemic changes in its constituent myocytes. Crucially, this propagation slowdown emerges in the simulation automatically, without requiring additional parameter adjustments to the intercellular interaction.

The second type of slowed conduction in ischemic myocardium is associated with a partial disruption and/or reduced conductance of intercellular gap junctions, which can occur during later stages of acute ischemia (≥15 min) [42]. In the 1D model, this form of conduction slowing is simulated by directly decreasing the electrodiffusion coefficient *D* (see Equation (4)).

This work aims to comparatively analyze the contribution of these two types of conduction-slowing mechanisms to the electrical function of ischemic myocardium. Therefore, we utilize two 1D models simulating 15 min ischemia:

- ***Model IM*1_15_**, in which the electrodiffusion coefficient *D* is maintained unchanged at the value for healthy myocardium (*D* = 300 mm^2^/s); the conduction slowing is caused solely by the intracellular ischemic changes in the cardiomyocytes.
- ***Model IM*2_15_**, in which the parameter *D* is reduced relative to its value in the healthy tissue model.

### 4.5. Safety Factor for Cardiac Propagation

The cardiac safety factor (SF) is a putative quantifier of the robustness of propagation in heart tissue [44]. It quantifies the surplus of current delivered to a cell relative to the amount required to depolarize the membrane to threshold. Several formulations of the SF have been proposed in the literature [5,23,44,45,46,47]. In particular, propagation fails when the SF is lower than 1, indicating that the excitation wave cannot spread downstream.

In this study, we employ the formulation by Shaw and Rudy [5] as an indicator of excitation block in myocardial tissue.

In its general form, the SF is given by the following expression:

(11) $$SF=\frac{C_{m}\Delta V+Q_{out}}{Q_{in}},$$

where *Q_in_* is the charge that the cell receives from the upstream tissue, and *Q_out_* is the charge that the cell generates for the depolarization of downstream cells, computed over the time of rise in the membrane voltage (*V*).

We use the following variant of Equation (11) to calculate *SF* for any cardiomyocyte in the strand:

(12) $$SF=\frac{\int_{t_{stim}}^{t_{V_{\max}}}I_{out}dt+\int_{t_{stim}}^{t_{V_{\max}}}I_{cell}dt}{\int_{t_{stim}}^{t_{V_{\max}}}I_{in}dt},$$

where *I_cell_* = −(1/*C_m_*)*∑I_ion_* is the capacitive current of the cell, *I_out_* is the intercellular current flowing out of the cell towards its downstream neighbor, and *I_in_* is the intercellular current entering the cell from its upstream neighbor (all currents normalized by the membrane capacitance, *C_m_*). The integration interval spans from the onset of cellular stimulation (*t_stim_*) to the point of maximum deviation from the resting potential (*t_Vmax_*).

## 5. Conclusions

This study employed human electromechanical myocardial models to investigate the roles of electrophysiological and mechanical mechanisms in conduction block during acute ischemia. Using our 1D model, we demonstrated:

1. The hyperkalemia-induced gradual reduction in the fast sodium current *i_Na_* in cardiomyocytes is the primary cause of conduction slowing and, ultimately, conduction block. This reduction is significantly amplified by mechano-electric and mechano-calcium feedback loops operating within the tissue.
2. A moderate, ischemia-induced reduction in gap junction coupling can partially or completely eliminate conduction block. We identified it as a potential compensatory mechanism.

Thus, our simulations highlight a possible counterintuitive dual role of conduction slowing during acute ischemia. Slowing caused by reduced *i_Na_* promotes the block, whereas slowing caused by moderate gap-junction uncoupling can prevent it.

In contrast to previous work on mathematical modeling that dealt more with the factors promoting the block, we focused on the underlying mechanisms, including not only electrical but also mechanical intercellular communication. Our findings underscore the critical importance of incorporating mechanical activity and its feedback on electrical processes for an integrative understanding of arrhythmogenesis in acute myocardial ischemia.

All the above conclusions are obtained using a particular mathematical model. In other words, they signify our theoretical predictions and cannot be considered to be completely established facts. Further studies combining both modeling and experimental approaches are necessary to assess the validity of these predictions.

## 6. Limitations

Several simplifications were adopted in the present study dealing with the contribution of the mechano-electrical feedback to conduction slowing and block during the early phases of the acute ischemia. In particular, we focused on the effects of hyperkalemia, ATP-sensitive K^+^ current, and partial gap junction uncoupling. These choices delimit the scope of our conclusions and define directions for subsequent work.

*Temporal scope and ATP-dependent active transport.* Our simulations target the early stages of acute regional ischemia (the first several minutes), during which hyperkalemia, mild acidosis and partial gap junction uncoupling progressively develop, but bulk ATP depletion has not yet substantially compromised active transport during the earliest stage (five-minute ischemia) [48,49]. Accordingly, the Na^+^/K^+^ ATPase and the SR Ca^2+^-ATPase (SERCA) were modeled with their baseline formulations, without explicit ATP-dependent down-regulation. We acknowledge that during the next early ischemic stages, a gradual decline in the ATP/ADP ratio begins to affect both pumps, with downstream consequences for intracellular Na^+^ and Ca^2+^ homeostasis and, indirectly, for the electrophysiological and mechanical behavior examined here. Therefore, in separate numerical experiments not involved in this work, we incorporated an explicit metabolic compartment with ATP-dependent regulation of the Na^+^/K^+^ pump and SERCA in addition to the activation of the ATP-sensitive K^+^ current and elevated extracellular [K^+^]*_o_* presented here. A contribution of the twofold-reduced Na^+^/K^+^ pump or SERCA activity due to the decrease in the ATP pool to the amplitude and time characteristics of both isometric force and AP in cardiomyocytes was observed in that series of simulations, but it did not exceed 10% relative to the respective effects of both ATP-sensitive K^+^ current and hyperkalemia. Therefore, we considered it possible to eliminate the influence of ATP on both pumps in this study. However, we will likely address these issues in the future for another particular problem, if even the moderate consequent mechanical and/or electrical effects of the reduced pump activity turn out to contribute significantly to the analyzed effects.

*Intracellular pH and acidosis.* The present model does not explicitly incorporate the effects of intracellular acidification, which is a well-established component of acute ischemia. A drop in intracellular pH is known to modulate several determinants of the action potential and of excitation–contraction coupling. In particular, acidification reduces the L-type Ca^2+^ current and the background inwardly rectifying K^+^ current, and decreases the Ca^2+^-binding affinity of the myofilaments, thereby altering Ca^2+^ buffering by the contractile apparatus [50,51]. Because the mechano-electrical pathway investigated here depends critically on intracellular Na^+^ and Ca^2+^ handling, pH-dependent modulation of these processes could quantitatively shift the thresholds at which the protective or pro-arrhythmic effects we report emerge. However, the qualitative structure of the mechanism observed in the model—namely, *i_Na_* reduction driven by hyperkalemia together with Ca^2+^ transients modulated by the mechanically interacting cardiomyocytes affecting, in turn, NCX activity in these myocytes—is unlikely to disappear due to the above-mentioned acidosis factors. Moreover, as we emphasized in the Section 3, acidosis-induced decrease in the sodium conductance does not exceed 25%, whereas the factors we did consider in the study reduced *i_Na_* by an order of magnitude, which turned out to be necessary and sufficient for the occurrence of an excitation block. To identify the mechanisms underlying these key factors, we had to eliminate other factors, including acidosis, from our simulations in the model. Of course, a dedicated pH-coupled model is required in further studies, in particular, to verify the quantitative threshold values for hyperkalemia that can trigger the block.

*One-dimensional representation of the myocardium tissue.* The one-dimensional strand (1D) model used in the study represents the minimal configuration capable of reproducing conduction block. Its primary limitation is the inability to capture complex arrhythmic substrates like reentry, which require two-dimensional (2D) or three-dimensional (3D) architecture for the wavefront to circulate around a functional obstacle. Therefore, while our model is well-suited for investigating the initiation of conduction block, it cannot be used to study the subsequent maintenance of arrhythmias via reentrant circuits.

*Tissue homogeneity in the model.* Our 1D strand was formed from a uniform cell type. Meantime, ventricular myocardium is known to be electrophysiologically and mechanically heterogeneous, both transmurally (epicardium, midmyocardium, endocardium) and in comparison between the right and left ventricles. As shown by Lukas & Antzelevitch [52,53], canine subepicardial and subendocardial cardiomyocytes differ in the density of the transient outward current (*i_to_*). During ischemia, this leads to loss of the action potential dome in subepicardial cells, but not in subendocardial cells. This heterogeneity of the I_to_ response to ischemia creates the basis for phase 2 reentry. In humans, similar transmural *i_to_* gradients also exist. Moreover, *i_to_* density is higher in the right ventricle than in the left [54,55]. Future studies using heterogeneous 1D/2D/3D models are needed to address these complexities. Extending the present framework to heterogeneous virtual samples in which epicardial, midmyocardial and endocardial zones are represented and respond differently to ischemia is a natural next step and is expected to reveal additional arrhythmogenic substrates that the present model does not account for.

*Other simplifications.* Beyond the points above, the model does not represent stretch-activated ion channels, autonomic modulation, or spatial gradients of extracellular [K^+^]*_o_* and pH within the ischemic zone or across the border zone. Each of these is known to contribute to ischemia-induced arrhythmogenesis and constitutes a meaningful target for future model extensions.

## Data Availability

The data presented in this study are available on request from the corresponding author. The data are not publicly available due to the ongoing nature of the research project.

## Supplementary Materials

The following supporting information can be downloaded at: https://www.mdpi.com/article/10.3390/ijms27146302/s1. The supplementary materials include a detailed description of the equations and parameters for the cardiomyocyte and tissue strand models.

## Abbreviations

The following abbreviations are used in this manuscript:

1D	One-dimensional
AP	Action potential
APD	Action potential duration
APD_90_	Action potential duration at 90% repolarization
ATP	Adenosine triphosphate
[*ATP*]*_i_*	Intracellular ATP concentration
Ca^2+^	Calcium ion
[Ca^2+^]*_i_*	Intracellular calcium concentration
CaTnC	Calcium-troponin C complex
*CE*	Contractile element
*C_m_*	Membrane capacitance
*D*	Electrodiffusion coefficient
*EO*	Ekaterinburg–Oxford (model)
F_CE_	Contractile element force
GJ	Gap junction
*g_K_*_1_	Maximal inward-rectifier K+ channel conductance
*g_KATP_*	Maximal ATP-sensitive K+ channel conductance
*g_Na_*	Maximal sodium channel conductance
*i_CaL_*	L-type calcium current
*i_K_*_1_	Inward rectifier potassium current
*i_KATP_*	ATP-sensitive potassium current
*i_Kr_*	Rapid delayed rectifier potassium current
*i_Ks_*	Slow delayed rectifier potassium current
*i_Na_*	Fast sodium current
*i_NaCa_*	Sodium–calcium exchanger current
*i_NaK_*	Na^+^–K^+^ pump current
*i_pCa_*	Sarcolemmal Ca^2+^ pump current
***IM*****1_15_**	Ischemia model 15 min (D unchanged)
***IM*****2_15_**	Ischemia model 15 min (D reduced)
K^+^	Potassium ion
[K^+^]*_o_*	Extracellular potassium concentration
Na^+^	Sodium ion
NCX	Sodium–calcium exchanger
*PE*	Parallel elastic element
RP	Resting potential
*SE*	Series elastic element
SERCA	Sarco/endoplasmic reticulum Ca^2+^-ATPase
*SF*	Safety factor
SR	Sarcoplasmic reticulum
SS	Subspace (dyadic cleft)
*TP+M*	ten Tusscher–Panfilov + mechanics (model)
*VS_x_*	Viscous element
XB	Cross-bridge
*XSE*	External series elastic element
