# Supplementary material for: Conduction Block in the Human Ischemic Myocardium: Insights from a 1D Electromechanical Model
Source: Int J Mol Sci. 2026 Jul 15;27(14):6302. doi: 10.3390/ijms27146302 (PMC13410128; doi:10.3390/ijms27146302)
Supplement: Supplementary file 1 [file ijms-27-06302-s001.zip › ijms-4299953-supplementary.pdf]

I. TP+M model

State variables

	Definition	Initial value	Unit
V	membrane potential	-85.86	mV
d	voltage-dependent activation gate (i_{CaL})	$3.102 \cdot 10^{-5}$	dimensionless
f_2	fast voltage-dependent inactivation gate (i_{CaL})	0.9995	dimensionless
$fCass$	intracellular Ca^{2+} inactivation gate (i_{CaL})	1.0	dimensionless
f	slow voltage-dependent inactivation gate (i_{CaL})	0.98	dimensionless
R	proportion of closed I_{rel} channels	0.9876	dimensionless
O	proportion of opened I_{rel} channels	0.0	dimensionless
I	proportion of inactivated I_{rel} channels	0.0	dimensionless
RI	proportion of resting inactivated I_{rel} channels	0.0124	dimensionless
Ca_{sr}	sarcoplasmic reticulum Ca^{2+} concentration	1.19	mM
Ca_{ss}	subspace Ca^{2+} concentration	0.00018	mM
$CaTnC$	Ca^{2+}-troponin C complexes concentration	0.00770	mM
h	fast inactivation gate (i_{Na})	0.7617	dimensionless
j	slow inactivation gate (i_{Na})	0.7602	dimensionless
m	activation gate (i_{Na})	0.0019	dimensionless
$Xr1$	activation gate (i_{Kr})	0.00019	dimensionless
$Xr2$	inactivation gate (i_{Kr})	0.4777	dimensionless
Xs	activation gate (i_{Ks})	0.0034	dimensionless
r	voltage-dependent activation gate (i_{to})	$2.1 \cdot 10^{-8}$	dimensionless
s	voltage-dependent inactivation gate (i_{to})	1.0	dimensionless
K_i	intracellular K^+ concentration	134.72	mM
Na_i	intracellular Na^+ concentration	11.31	mM
Ca_i	intracellular Ca^{2+} concentration	0.000052	mM
l_1	deformation of CE against its slack length	0.392	μm
l_2	deformation of PE against its slack length	0.392	μm
l_3	deformation of XSE against its slack length	0.052	μm
v	velocity of CE deformation	0	μm/ms
w	velocity of PE deformation	0	μm/ms
N	cross-bridges concentration	$4.58 \cdot 10^{-6}$	dimensionless

Constants

	Definition	Value	Unit
$stim_{amp}$	amplitude of i_{stim}	52	pA/pF
$stim_{dur}$	duration of i_{stim}	1	ms
$stim_{per}$	periodicity of i_{stim}	1000	ms
$stim_{start}$	start of i_{stim}	10	ms
F	Faraday constant	96485.3415	C/M

Constants (continued)

	Definition	Value	Unit
R	gas constant	8314.472	$\mathrm{mJ \cdot K^{-1} \cdot M^{-1}}$
T	temperature	310	K
Cm	cell capacitance	0.185	μF
V_c	cytoplasmic volume	0.016404	$\mathrm{mm^3}$
V_{sr}	sarcoplasmic reticulum volume	0.001094	$\mathrm{mm^3}$
V_{ss}	subspace volume	0.0000547	$\mathrm{mm^3}$
Ca_o	intracellular Ca^{2+} concentration	2	mM
K_o	extracellular K^+ concentration	5.4	mM
Na_o	extracellular Na^+ concentration	140	mM
g_{CaL}	maximal i_{CaL} conductance	0.00006	l/(F· s)
g_{bCa}	maximal i_{bCa} conductance	0.000592	nS/pF
K_{pCa}	Ca_i half-saturation constant of i_{pCa}	0.0005	mM
g_{pCa}	maximal i_{pCa} conductance	0.2476	pA/pF
Buf_{sr}	total sarcoplasmic buffer concentration	10	mM
Buf_{ss}	total subspace buffer concentration	0.4	mM
Buf_c	total (except $CaTnC$) cytoplasmic buffer concentration	0.13	mM
EC	Ca_{sr} half-saturation constant of k_{casr}	1.5	mM
$K_{buf_{sr}}$	Ca_{sr} half-saturation constant for sarcoplasmic buffer	0.3	mM
$K_{buf_{ss}}$	Ca_{SS} half-saturation constant for subspace buffer	0.00025	mM
K_{buf_c}	Ca_i half-saturation constant for cytoplasmic buffer	0.00085	mM
V_{leak}	maximal I_{leak} conductance	0.00036	$\mathrm{ms^{-1}}$
V_{rel}	maximal I_{rel} conductance	0.1224	$\mathrm{ms^{-1}}$
V_{xfer}	maximal I_{xfer} conductance	0.00456	$\mathrm{ms^{-1}}$
K_{up}	half-saturation constant of I_{up}	0.00025	mM
V_{maxup}	maximal I_{up} conductance	0.00765	mM/ms
$k1_{prime}$	R to O and RI to I I_{rel} transition rate	2.1	$\mathrm{mM^{-2} \cdot ms^{-1}}$
$k2_{prime}$	O to I and R to RI I_{rel} transition rate	0.025	$\mathrm{mM^{-1} \cdot ms^{-1}}$
$k3$	O to I and R to RI I_{rel} transition rate	0.06	$\mathrm{ms^{-1}}$
$k4$	I to O and RI to I I_{rel} transition rate	0.005	$\mathrm{ms^{-1}}$
max_{sr}	maximum value of k_{casr}	2.5	dimensionless
min_{sr}	minimum value of k_{casr}	1	dimensionless
Π_{min}	parameter of Π_{N_A} function	0.02	dimensionless
s_c	parameter of $N_A(CaTnC, N)$ function	1.0	dimensionless
TnC_{tot}	total concentration of TnC	0.07	mM
k_A	cooperativity parameter	28.0	$\mathrm{mM^{-1}}$
a_{off}	maximum rate constant for $CaTnC$ dissociation	0.17	$\mathrm{ms^{-1}}$
a_{on}	rate constant for $CaTnC$ association	35.0	$\mathrm{mM^{-1} \cdot ms^{-1}}$
g_{Na}	maximal i_{Na} conductance	14.838	nS/pF

Constants (continued)

	Definition	Value	Unit
g_{bna}	maximal i_{bNa} conductance	0.00029	nS/pF
g_{K1}	maximal i_{K1} conductance	5.405	nS/pF
g_{pK}	maximal i_{pK} conductance	0.0146	nS/pF
g_{Kr}	maximal i_{Kr} conductance	0.153	nS/pF
P_{kna}	relative i_{Ks} permeability to Na^+	0.03	dimensionless
g_{Ks}	maximal i_{Ks} conductance	0.392	nS/pF
g_{to}	maximal i_{to} conductance	0.735	nS/pF
K_{NaCa}	maximal i_{NaCa}	10000	pA/pF
K_{sat}	saturation factor for i_{NaCa}	0.1	dimensionless
Km_{Ca}	Ca_i half-saturation constant for i_{NaCa}	1.38	mM
Km_{Nai}	Na_i half-saturation constant for i_{NaCa}	87.5	mM
α	factor enhancing outward nature of i_{NaCa}	1	dimensionless
γ	voltage dependence parameter of i_{NaCa}	0.35	dimensionless
K_{mNa}	Na_i half-saturation constant for i_{NaK}	40	mM
K_{mK}	K_o half-saturation constant for i_{NaK}	1	mM
P_{NaK}	maximal i_{NaK}	2.724	pA/pF
λ	scale parameter of F_{CE}	250.0	AFU
α_1	exponential coefficient of F_{SE}	14.6	μm^{-1}
β_1	linear coefficient of F_{SE}	4.2	AFU
α_2	exponential coefficient of F_{PE}	14.6	μm^{-1}
β_2	linear coefficient of F_{PE}	0.009	AFU
α_3	exponential coefficient of F_{XSE}	55.0	μm^{-1}
β_3	linear coefficient of F_{XSE}	0.11	AFU
α_{vp_l}	exponential coefficient of F_{VS_1}	16.0	μm^{-1}
α_{vp_s}	exponential coefficient of F_{VS_1}	16.0	μm^{-1}
β_{vp_l}	linear coefficient of F_{VS_1}	0.1	AFU$\cdot$ ms/μm
β_{vp_s}	linear coefficient of F_{VS_1}	10	AFU$\cdot$ ms/μm
α_{vs_l}	exponential coefficient of F_{VS_2}	46.0	μm^{-1}
α_{vs_s}	exponential coefficient of F_{VS_2}	39.0	μm^{-1}
β_{vs_l}	linear coefficient of F_{VS_2}	20.0	AFU$\cdot$ ms/μm
β_{vs_s}	linear coefficient of F_{VS_2}	60.0	AFU$\cdot$ ms/μm
v_{max}	parameter of p function	0.0055	μm/ms
a	parameter of p function	0.25	dimensionless
d_h	parameter of P_{star} function	0.5	dimensionless
α_P	parameter of G_{star} function	4.0	dimensionless
α_G	parameter of G_{star} function	1.0	dimensionless
k_μ	parameter of M_A function	0.6	dimensionless
μ	parameter of M_A function	3.3	dimensionless
g_1	parameter of n_1 function	0.6	μm^{-1}
g_2	parameter of n_1 function	0.52	dimensionless
$n1_A$	parameter of n_1 function	0.5	dimensionless
$n1_B$	parameter of n_1 function	55	μm
$n1_C$	parameter of n_1 function	1	dimensionless
$n1_Q$	parameter of n_1 function	0.835	dimensionless
$n1_K$	parameter of n_1 function	1	dimensionless

Constants (continued)

	Definition	Value	Unit
$n1_\nu$	parameter of n_1 function	5	dimensionless
S_0	parameter of L_{oz} function	1.14	μm
S_{055}	parameter of L_{oz} function	0.55	μm
S_{046}	parameter of L_{oz} function	0.46	μm
κ_0	parameter of κ function	2.1	dimensionless
κ_1	parameter of κ function	0.55	dimensionless
κ_2	parameter of κ function	0.0	dimensionless
m_0	fraction of strongly attached Xb in steady state isometric conditions	0.9	dimensionless
q_1	parameter of q function	0.0173	ms^{-1}
q_2	parameter of q function	0.259	ms^{-1}
q_3	parameter of q function	0.0173	ms^{-1}
q_4	parameter of q function	0.015	ms^{-1}
α_Q	parameter of q function	10.0	dimensionless
β_Q	parameter of q function	5.0	dimensionless
x_{st}	parameter of q function	0.964285	dimensionless
$r0$	preload	2.552	AFU (for $L_{init} = 90\% L_{max}$)
F_{aft}	afterload	6.89	AFU (for $10\% F_{isom}$)
k_{phys_rel}	parameter of V_{phys_rel} function	0.05	ms^{-1}
a_{phys_rel}	parameter of V_{phys_rel} function	*calculated*	μm
t_{phys_rel}	parameter of V_{phys_rel} function	*calculated*	ms
per_{phys_rel}	parameter of V_{phys_rel} function	230	ms

i_{stim}, stimulating current.

Calcium currents:
i_{CaL}, L-type Ca^{2+} current;
i_{bCa}, background Ca^{2+} current.

Calcium translocations:
I_{rel}, Ca^{2+} release from the sarcoplasmic reticulum (SR) via ryanodine receptors to the subspace (SS);
I_{xfer}, Ca^{2+} diffusion from SS to the cytoplasm (C);
I_{leak}, a small Ca^{2+} leakage from the SR to the cytoplasm;
I_{up}, Ca^{2+} pumping from the cytoplasm to the SR.
O, open conducting state of I_{rel}; R, resting closed state of I_{rel}; I, inactivated closed state of I_{rel}; RI, resting inactivated closed state of I_{rel}.

Calcium buffers:
CaB, buffering by other than $CaTnC$ intracellular ligands;
$CaTnC$, Ca^{2+}-troponin C complexes complexes;
Π_{N_A}, dependence defining cooperativity of the contractile proteins;
N_A, average fraction of the attached cross-bridges per one $CaTnC$ complex;
$CaSRB$, calcium buffering in SR;
$CaSSB$, subspace calcium buffering.

Sodium currents:
i_{Na}, fast Na^+ current;
i_{bNa}, background Na^+ current.

Potassium currents:
i_{K1}, inward rectifier K^+ current;
i_{to}, transient outward current;
i_{Kr}, i_{Ks}, rapid and slow delayed rectifier current;
i_{pK}, plateau K^+ current.

Pumps and exchangers:
i_{pCa}, sarcolemmal Ca^{2+} pump current;
i_{NaCa}, Na^{+}˘Ca^{2+} exchanger current;
i_{NaK}, Na^{+}˘K^{+} pump current.

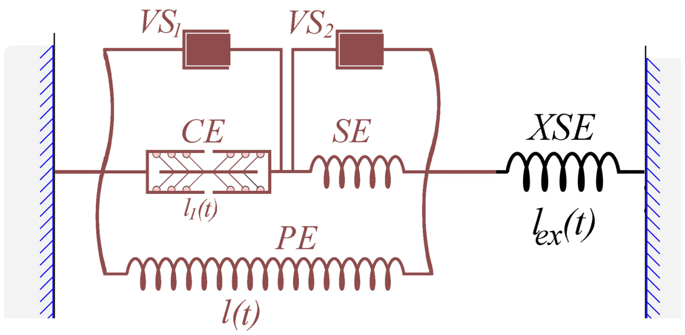

Figure S1: Rheological scheme in the TP+M model

Forces: AFU - arbitrary force unit;
F_{CE}, contractile element (sarcomere) (CE) force;
F_{SE}, serial elastic element (SE) force;
F_{PE}, parallel elastic element (PE) force;
F_{XSE}, extra serial elastic element (XSE) force;
F_{VS_1}, F_{VS_2}, viscous elements ($VS1$, $VS2$) forces.
l_1, deviation of contractile length from its slack length.
l_2, deviation of parallel elastic element length from its slack length.
l_3, deviation of extra series element length from its slack length.
$l = l_2 + l_3$, deviation of the sample length from its slack length.
p, dependence of the average cross-bridge force on the sarcomere shortening/lengthening velocity.
P_{star}, dependence of the steady-state sarcomere force on the sarcomere shortening/lengthening velocity.
G_{star}, dependence of the steady-state sarcomere stiffness on the velocity.
M_A, means end-to-end interaction between adjacent tropomyosin segments in the case if both of them affected by the respective $CaTnC$ complexes formation.
n_1, probability of that a myosin head can 'find' a vacant site on the actin filament.

L_{oz}, instantaneous length of thick and thin filament overlap zone.
κ, function required for variation the ratio between rates of cross-bridge attachment and detachment.
q, stationary relation 'stiffness-velocity' for the sample.
L_{init}, initial length of the sample.
L_{max}, corresponds to a sarcomere length equal to 2.23 μm.
F_{isom}, maximum of isometric force at given L_{init}.
V_{phys_rel}, dependence of sample length return during physiological relaxation.
k_{phys_rel}, physiological relaxation velocity.
a_{phys_rel}, physiological relaxation amplitude calculated as a difference between end-systolic and initial lengths of the sample.
t_{phys_rel}, time to start physiological relaxation fixed at the moment when $F_{sample} = r0$.
per_{phys_rel} period of physiological relaxation.

MODEL EQUATIONS

TNNP BLOCK (with modifications)

MEMBRANE POTENTIAL

$$i_{Stim} = \begin{cases} -stim_{amp} & \text{if } \left(time - \lfloor \frac{time}{stim_{per}} \rfloor \cdot stim_{per} \geq stim_{start}\right) \text{ and} \\ & \text{and } \left(time - \lfloor \frac{time}{stim_{per}} \rfloor \cdot stim_{per} \leq stim_{start} + stim_{dur}\right) \\ 0 & \text{otherwise} \end{cases}$$

$$\frac{\mathrm{d}V}{\mathrm{d}time} = \frac{-1}{Cm} \cdot (i_{K1} + i_{to} + i_{Kr} + i_{Ks} + i_{CaL} + i_{NaK} + i_{Na} + i_{b_{Na}} + i_{NaCa} + i_{b_{Ca}} + i_{p_K} + i_{p_{Ca}} + i_{Stim})$$

REVERSAL POTENTIALS

$$E_{Na} = \frac{R \cdot T}{F} \cdot \ln \frac{Na_o}{Na_i}$$

$$E_K = \frac{R \cdot T}{F} \cdot \ln \frac{K_o}{K_i}$$

$$E_{Ks} = \frac{R \cdot T}{F} \cdot \ln \frac{K_o + P_{kna} \cdot Na_o}{K_i + P_{kna} \cdot Na_i}$$

$$E_{Ca} = \frac{0.5 \cdot R \cdot T}{F} \cdot \ln \frac{Ca_o}{Ca_i}$$

L-TYPE Ca^{2+} CURRENT

$$i_{CaL} = g_{CaL} \cdot d \cdot f \cdot f_2 \cdot f_{Cass} \cdot 4 \cdot \frac{(V-15) \cdot F^2}{R \cdot T} \cdot \frac{\left(0.25 \cdot Ca_{ss} \cdot e^{\frac{2 \cdot (V-15) \cdot F}{R \cdot T}} - Ca_o\right)}{e^{\frac{2 \cdot (V-15) \cdot F}{R \cdot T}} - 1}$$

L-TYPE Ca^{2+} CURRENT. d GATE

$$d_{inf} = \frac{1}{1 + e^{\frac{-8-V}{7.5}}}$$

$$\alpha_d = \frac{1.4}{1 + e^{\frac{-35-V}{13}}} + 0.25$$

$$\beta_d = \frac{1.4}{1 + e^{\frac{V+5}{5}}}$$

$$\gamma_d = \frac{1}{1 + e^{\frac{50-V}{20}}}$$

$$\tau_d = 1 \cdot \alpha_d \cdot \beta_d + \gamma_d$$

$$\frac{\mathrm{d}d}{\mathrm{d}time} = \frac{d_{inf} - d}{\tau_d}$$

L-TYPE Ca^{2+} CURRENT. $f2$ GATE

$$f2_{inf} = \frac{0.67}{1 + e^{\frac{V+35}{7}}} + 0.33$$

$$\tau_{f2} = 562 \cdot e^{\frac{-(V+27)^2}{240}} + \frac{31}{1 + e^{\frac{25-V}{10}}} + \frac{80}{1 + e^{\frac{V+30}{10}}}$$

$$\frac{\mathrm{d}f_2}{\mathrm{d}time} = \frac{f2_{inf} - f2}{\tau_{f2}}$$

L-TYPE Ca^{2+} CURRENT. fCa_{SS} GATE

$$fCass_{inf} = \frac{0.6}{1 + \left(\frac{Ca_{ss}}{0.05}\right)^2} + 0.4$$

L-TYPE Ca^{2+} CURRENT. fCa_{SS} GATE (CONTINUED)

$$\tau_{fCass} = \frac{80}{1 + \left(\frac{Ca_{ss}}{0.05}\right)^2} + 2$$

$$\frac{\mathrm{d}f_{Cass}}{\mathrm{d}time} = \frac{fCass_{inf} - fCass}{\tau_{fCass}}$$

L-TYPE Ca^{2+} CURRENT. f GATE

$$f_{inf} = \frac{1}{1 + e^{\frac{V+20}{7}}}$$

$$\tau_f = 1102.5 \cdot e^{\frac{-(V+27)^2}{225}} + \frac{200}{1 + e^{\frac{13-V}{10}}} + \frac{180}{1 + e^{\frac{V+30}{10}}} + 20$$

$$\frac{\mathrm{d}f}{\mathrm{d}time} = \frac{f_{inf} - f}{\tau_f}$$

Ca^{2+} BACKGROUND CURRENT

$$i_{b_{Ca}} = g_{bca} \cdot (V - E_{Ca})$$

Ca^{2+} PUMP CURRENT

$$i_{p_{Ca}} = \frac{g_{pCa} \cdot Ca_i}{Ca_i + K_{pCa}}$$

Ca^{2+}-INDUCED-Ca^{2+} RELEASE FLOW (i_{rel})

$$kcasr = max_{sr} - \frac{max_{sr} - min_{sr}}{1 + \left(\frac{EC}{Ca_{sr}}\right)^2}$$

$$k1 = \frac{k1_{prime}}{kcasr}$$

$$k2 = k2_{prime} \cdot kcasr$$

$$\frac{\mathrm{d}R}{\mathrm{d}time} = (k4 \cdot RI - k2 \cdot R \cdot Ca_{ss}) - (k1 \cdot R \cdot (Ca_{ss})^2 - k3 \cdot O)$$

$$\frac{\mathrm{d}O}{\mathrm{d}time} = (k1 \cdot R \cdot (Ca_{ss})^2 - k3 \cdot O) - (k2 \cdot O \cdot Ca_{ss} - k4 \cdot I)$$

$$\frac{\mathrm{d}I}{\mathrm{d}time} = (k2 \cdot O \cdot Ca_{ss} - k4 \cdot I) - (k3 \cdot I - k1 \cdot RI \cdot (Ca_{ss})^2)$$

$$\frac{\mathrm{d}RI}{\mathrm{d}time} = (k3 \cdot I - k1 \cdot RI \cdot (Ca_{ss})^2) - (k4 \cdot RI - k2 \cdot R \cdot Ca_{ss})$$

$$i_{rel} = V_{rel} \cdot O \cdot (Ca_{sr} - Ca_{ss})$$

Ca^{2+} DYNAMICS

$$i_{up} = \frac{Vmax_{up}}{1 + \frac{K_{up}^2}{Ca_i^2}}$$

$$i_{leak} = V_{leak} \cdot (Ca_{sr} - Ca_i)$$

$$i_{xfer} = V_{xfer} \cdot (Ca_{ss} - Ca_i)$$

$$B_{Cabufc} = \frac{1}{1 + \frac{Buf_c \cdot K_{buf_c}}{(Ca_i + K_{buf_c})^2}}$$

$$B_{Cabufsr} = \frac{1}{1 + \frac{Buf_{sr} \cdot K_{buf_{sr}}}{(Ca_{sr} + K_{buf_{sr}})^2}}$$

$$B_{Cabufss} = \frac{1}{1 + \frac{Buf_{ss} \cdot K_{buf_{ss}}}{(Ca_{ss} + K_{buf_{ss}})^2}}$$

$$N_A = \frac{TnC_{tot} \cdot N \cdot sc}{L_{oz} \cdot CaTnC}$$

$$\Pi_{N_A} = \begin{cases} 1 & \text{if } N_A \leq 0 \\ \Pi_{min}^{N_A} & \text{if } N_A \leq 1 \\ \Pi_{min} & \text{otherwise} \end{cases}$$

$$\frac{\mathrm{d}CaTnC}{\mathrm{d}time} = a_{on} \cdot (TnC_{tot} - CaTnC) \cdot Ca_i - a_{off} \cdot e^{-k_A \cdot CaTnC} \cdot \Pi_{N_A} \cdot CaTnC$$

$$\frac{\mathrm{d}Ca_{sr}}{\mathrm{d}time} = B_{Cabufsr} \cdot (i_{up} - (i_{rel} + i_{leak}))$$

$$\frac{\mathrm{d}Ca_{ss}}{\mathrm{d}time} = B_{Cabufss} \cdot \left(\frac{-1 \cdot i_{CaL} \cdot Cm}{2 \cdot 1 \cdot V_{ss} \cdot F} + \frac{i_{rel} \cdot V_{sr}}{V_{ss}} - \frac{i_{xfer} \cdot V_c}{V_{ss}}\right)$$

$$CaB = \frac{Buf_c \cdot Ca_i}{Ca_i + K_{buf_c}}$$

$$CaSRB = \frac{Buf_{sr} \cdot Ca_{sr}}{Ca_{sr} + K_{buf_{sr}}}$$

$$CaSSB = \frac{Buf_{ss} \cdot Ca_{ss}}{Ca_{ss} + K_{buf_{ss}}}$$

FAST Na^+ CURRENT

$$i_{Na} = g_{Na} \cdot m^3 \cdot h \cdot j \cdot (V - E_{Na})$$

FAST Na^+ CURRENT. h GATE

$$h_{inf} = \frac{1}{\left(1 + e^{\frac{V+71.55}{7.43}}\right)^2}$$

$$\alpha_h = \begin{cases} 0.057 \cdot e^{\frac{-(V+80)}{6.8}} & \text{if } V < -40 \\ 0 & \text{otherwise} \end{cases}$$

$$\beta_h = \begin{cases} 2.7 \cdot e^{0.079 \cdot V} + 310000 \cdot e^{0.3485 \cdot V} & \text{if } V < -40 \\ \dfrac{0.77}{0.13 \cdot \left(1 + e^{\frac{V+10.66}{-11.1}}\right)} & \text{otherwise} \end{cases}$$

$$\tau_h = \frac{1}{\alpha_h + \beta_h}$$

$$\frac{\mathrm{d}h}{\mathrm{d}time} = \frac{h_{inf} - h}{\tau_h}$$

FAST Na^+ CURRENT. j GATE

$$j_{inf} = \frac{1}{\left(1 + e^{\frac{V+71.55}{7.43}}\right)^2}$$

$$\alpha_j = \begin{cases} \dfrac{\left(-25428 \cdot e^{0.2444 \cdot V} - 6.948 \cdot 10^{-6} \cdot e^{-0.04391 \cdot V}\right) \cdot (V + 37.78)}{1 + e^{0.311 \cdot (V+79.23)}} & \text{if } V < -40 \\ 0 & \text{otherwise} \end{cases}$$

FAST Na^+ CURRENT. j GATE (CONTINUED)

$$\beta_j = \begin{cases} \dfrac{0.02424 \cdot e^{-0.01052 \cdot V}}{1 + e^{-0.1378 \cdot (V + 40.14)}} & \text{if } V < -40 \\ \dfrac{0.6 \cdot e^{0.057 \cdot V}}{1 + e^{-0.1 \cdot (V + 32)}} & \text{otherwise} \end{cases}$$

$$\tau_j = \frac{1}{\alpha_j + \beta_j}$$

$$\frac{\mathrm{d}j}{\mathrm{d}time} = \frac{j_{inf} - j}{\tau_j}$$

FAST Na^+ CURRENT. m GATE

$$m_{inf} = \frac{1}{\left(1 + e^{\frac{-56.86 - V}{9.03}}\right)^2}$$

$$\alpha_m = \frac{1}{1 + e^{\frac{-60 - V}{5}}}$$

$$\beta_m = \frac{0.1}{1 + e^{\frac{V + 35}{5}}} + \frac{0.1}{1 + e^{\frac{V - 50}{200}}}$$

$$\tau_m = 1 \cdot \alpha_m \cdot \beta_m$$

$$\frac{\mathrm{d}m}{\mathrm{d}time} = \frac{m_{inf} - m}{\tau_m}$$

Na^+ BACKGROUND CURRENT

$$i_{b_{Na}} = g_{bna} \cdot (V - E_{Na})$$

INWARD RECTIFIER K^+ CURRENT

$$\alpha_{K1} = \frac{0.1}{1 + e^{0.06 \cdot (V - E_K - 200)}}$$

$$\beta_{K1} = \frac{3 \cdot e^{0.0002 \cdot (V - E_K + 100)} + e^{0.1 \cdot (V - E_K - 10)}}{1 + e^{-0.5 \cdot (V - E_K)}}$$

$$xK1_{inf} = \frac{\alpha_{K1}}{\alpha_{K1} + \beta_{K1}}$$

$$i_{K1} = g_{K1} \cdot xK1_{inf} \cdot \sqrt{\frac{K_o}{5.4}} \cdot (V - E_K)$$

K^+ PLATEAU CURRENT

$$i_{p_K} = \frac{g_{pK} \cdot (V - E_K)}{1 + e^{\frac{25-V}{5.98}}}$$

RAPID TIME DEPENDENT K^+ CURRENT

$$i_{Kr} = g_{Kr} \cdot \sqrt{\frac{K_o}{5.4}} \cdot Xr1 \cdot Xr2 \cdot (V - E_K)$$

RAPID TIME DEPENDENT K^+ CURRENT. XR1 GATE

$$xr1_{inf} = \frac{1}{1 + e^{\frac{-26-V}{7}}}$$

$$\alpha_{xr1} = \frac{450}{1 + e^{\frac{-45-V}{10}}}$$

$$\beta_{xr1} = \frac{6}{1 + e^{\frac{V+30}{11.5}}}$$

$$\tau_{xr1} = 1 \cdot \alpha_{xr1} \cdot \beta_{xr1}$$

$$\frac{\mathrm{d}Xr1}{\mathrm{d}time} = \frac{xr1_{inf} - Xr1}{\tau_{xr1}}$$

RAPID TIME DEPENDENT K^+ CURRENT. XR2 GATE

$$xr2_{inf} = \frac{1}{1 + e^{\frac{V+88}{24}}}$$

$$\alpha_{xr2} = \frac{3}{1 + e^{\frac{-60-V}{20}}}$$

$$\beta_{xr2} = \frac{1.12}{1 + e^{\frac{V-60}{20}}}$$

$$\tau_{xr2} = 1 \cdot \alpha_{xr2} \cdot \beta_{xr2}$$

$$\frac{\mathrm{d}Xr2}{\mathrm{d}time} = \frac{xr2_{inf} - Xr2}{\tau_{xr2}}$$

SLOW TIME DEPENDENT K^+ CURRENT

$$i_{Ks} = g_{Ks} \cdot Xs^2 \cdot (V - E_{Ks})$$

SLOW TIME DEPENDENT K^+ CURRENT. XS GATE

$$xs_{inf} = \frac{1}{1 + e^{\frac{-5-V}{14}}}$$

$$\alpha_{xs} = \frac{1400}{\sqrt{1 + e^{\frac{5-V}{6}}}}$$

$$\beta_{xs} = \frac{1}{1 + e^{\frac{V-35}{15}}}$$

$$\tau_{xs} = 1 \cdot \alpha_{xs} \cdot \beta_{xs} + 80$$

$$\frac{\mathrm{d}Xs}{\mathrm{d}time} = \frac{xs_{inf} - Xs}{\tau_{xs}}$$

TRANSIENT OUTWARD K^+ CURRENT

$$i_{to} = g_{to} \cdot r \cdot s \cdot (V - E_K)$$

TRANSIENT OUTWARD K^+ CURRENT. R GATE

$$r_{inf} = \frac{1}{1 + e^{\frac{20-V}{6}}}$$

$$\tau_r = 9.5 \cdot e^{\frac{-(V+40)^2}{1800}} + 0.8$$

$$\frac{\mathrm{d}r}{\mathrm{d}time} = \frac{r_{inf} - r}{\tau_r}$$

TRANSIENT OUTWARD K^+ CURRENT. S GATE

$$s_{inf} = \frac{1}{1 + e^{\frac{V+20}{5}}}$$

$$\tau_s = 85 \cdot e^{\frac{-(V+45)^2}{320}} + \frac{5}{1 + e^{\frac{V-20}{5}}} + 3$$

$$\frac{\mathrm{d}s}{\mathrm{d}time} = \frac{s_{inf} - s}{\tau_s}$$

Na^+-Ca^{2+} EXCHANGER CURRENT

$$i_{NaCa} = \frac{K_{NaCa} \cdot \left(e^{\frac{\gamma \cdot V \cdot F}{R \cdot T}} \cdot Na_i^3 \cdot Ca_o - e^{\frac{(\gamma-1) \cdot V \cdot F}{R \cdot T}} \cdot Na_o^3 \cdot Ca_i \cdot \alpha \right)}{\left(Km_{Nai}^3 + Na_o^3 \right) \cdot (Km_{Ca} + Ca_o) \cdot \left(1 + K_{sat} \cdot e^{\frac{(\gamma-1) \cdot V \cdot F}{R \cdot T}} \right)}$$

Na^+-K^+ PUMP CURRENT

$$i_{NaK} = P_{NaK} \cdot \frac{K_o \cdot Na_i}{(K_o + K_{mk}) \cdot (Na_i + K_{mNa}) \cdot (1 + 0.1245 \cdot e^{\frac{-0.1 \cdot V \cdot F}{R \cdot T}} + 0.0353 \cdot e^{\frac{-V \cdot F}{R \cdot T}})}$$

Ca^{2+} DYNAMICS

$$\frac{\mathrm{d}Ca_i}{\mathrm{d}t} = B_{Cabufc} \cdot \left(\frac{(i_{leak} - i_{up}) \cdot V_{sr}}{V_c} + i_{xfer} - \frac{(i_{b_{Ca}} + i_{p_{Ca}} - 2 \cdot i_{NaCa}) \cdot Cm}{V_c \cdot F} - \frac{\mathrm{d}CaTnC}{\mathrm{d}t} \right)$$

K^+ DYNAMICS

$$\frac{\mathrm{d}K_i}{\mathrm{d}time} = \frac{-1 \cdot (i_{K1} + i_{to} + i_{Kr} + i_{Ks} + i_{pK} + i_{Stim} - 2 \cdot i_{NaK})}{1 \cdot V_c \cdot F} \cdot Cm$$

Na^+ DYNAMICS

$$\frac{\mathrm{d}Na_i}{\mathrm{d}time} = \frac{-1 \cdot (i_{Na} + i_{b_{Na}} + 3 \cdot i_{NaK} + 3 \cdot i_{NaCa})}{1 \cdot V_c \cdot F} \cdot Cm$$

MECHANICAL BLOCK

FORCE

$$F_{CE} = \lambda \cdot p_v \cdot N$$

$$F_{SE} = \beta_1 \cdot \left(e^{\alpha_1 \cdot (l_2 - l_1)} - 1\right)$$

$$F_{PE} = \beta_2 \cdot \left(e^{\alpha_2 \cdot l_2} - 1\right)$$

$$F_{XSE} = \beta_3 \cdot \left(e^{\alpha_3 \cdot l_3} - 1\right)$$

$$F_{VS_1} = k_{P_{vis}} \cdot v$$

$$F_{VS_2} = k_{S_{vis}} \cdot (w - v)$$

$$F_{sample} = F_{XSE}$$

CONTRACTION MODES

$$contraction_{mode} = \begin{cases} isometry & \\ isotony & \text{if } (F_{sample} > F_{aft}) \text{ and } \left(l \leq l_0 \cdot \left(1 + 1 \cdot 10^{-4}\right)\right) \\ isometric\_relaxation & \text{if } (l\ is\ end - systolic\ length) \text{ and } (F_{sample} > r0) \\ physiological\_relaxation & \text{starts after isometric relaxation when } (F_{sample} \leq r0) \end{cases}$$

$$l = l_2 + l_3$$

$$\frac{\mathrm{d}l_1}{\mathrm{d}time} = v$$

$$\frac{\mathrm{d}l_2}{\mathrm{d}time} = w$$

$$V_{phys\_rel} = \frac{a_{phys\_rel} \cdot k_{phys\_rel} \cdot e^{(-k_{phys\_rel} \cdot (t - (t_{phys\_rel} + per_{phys\_rel}/2)))}}{(1 + e^{(-k_{phys\_rel} \cdot (t - (t_{phys\_rel} + per_{phys\_rel}/2)))})^2}$$

$$\frac{\mathrm{d}l_3}{\mathrm{d}time} = \begin{cases} -w & \text{if } contraction_{mode} = isometry\ and\ isometric\_relaxation \\ 0 & \text{if } contraction_{mode} = isotony \\ V_{phys\_rel} - w & \text{if } contraction_{mode} = physiological\_relaxation \end{cases}$$

$$alp_p = \begin{cases} \alpha_{vp_l} & \text{if } v \leq 0 \\ \alpha_{vp_s} & \text{otherwise} \end{cases}$$

$$k_{P_{vis}} = \begin{cases} \beta_{vp_l} \cdot e^{\alpha_{vp_l} \cdot l_1} & \text{if } v \leq 0 \\ \beta_{vp_s} \cdot e^{\alpha_{vp_s} \cdot l_1} & \text{otherwise} \end{cases}$$

$$\phi_\chi = \begin{cases} \frac{-\left(\lambda \cdot K_\kappa \cdot p_v + alp_p \cdot k_{P_{vis}} \cdot v^2 + \left(\alpha_2 \cdot \beta_2 \cdot e^{\alpha_2 \cdot l_2} + \alpha_3 \cdot \beta_3 \cdot e^{\alpha_3 \cdot l_3}\right) \cdot w\right)}{\lambda \cdot N \cdot p_{prime_v} + k_{P_{vis}}}, \\ \quad \text{if } contraction_{mode} = isometry\ and\ isometric\_relaxation \\ \frac{-\left(\lambda \cdot K_\kappa \cdot p_v + alp_p \cdot k_{P_{vis}} \cdot v^2 + \alpha_2 \cdot \beta_2 \cdot e^{\alpha_2 \cdot l_2} \cdot w\right)}{\lambda \cdot N \cdot p_{prime_v} + k_{P_{vis}}}, \\ \quad \text{if } contraction_{mode} = isotonic \\ \frac{-\left(\lambda \cdot K_\kappa \cdot p_v + alp_p \cdot k_{P_{vis}} \cdot v^2 + \alpha_2 \cdot \beta_2 \cdot e^{\alpha_2 \cdot l_2} \cdot w - \alpha_3 \cdot \beta_3 \cdot e^{\alpha_3 \cdot l_3} \cdot \left(V_{phys\_rel} - w\right)\right)}{\lambda \cdot N \cdot p_{prime_v} + k_{P_{vis}}}, \\ \quad \text{if } contraction_{mode} = physiological\_relaxation \end{cases}$$

$$\frac{\mathrm{d}v}{\mathrm{d}time} = \phi_\chi$$

$$alp_s = \begin{cases} \alpha_{vs_l} & \text{if } w \leq v \\ \alpha_{vs_s} & \text{otherwise} \end{cases}$$

$$k_{S_{vis}} = \begin{cases} \beta_{vs_l} \cdot e^{\alpha_{vs_l} \cdot (l_2 - l_1)} & \text{if } w \leq v \\ \beta_{vs_s} \cdot e^{\alpha_{vs_s} \cdot (l_2 - l_1)} & \text{otherwise} \end{cases}$$

$$\frac{\mathrm{d}w}{\mathrm{d}time} = \begin{cases} \phi_\chi - alp_s \cdot (w - v)^2 - \dfrac{\alpha_1 \cdot \beta_1 \cdot e^{\alpha_1 \cdot (l_2 - l_1)} \cdot (w - v) + \left(\alpha_2 \cdot \beta_2 \cdot e^{\alpha_2 \cdot l_2} + \alpha_3 \cdot \beta_3 \cdot e^{\alpha_3 \cdot l_3}\right) \cdot w}{k_{S_{vis}}}, \\ \text{if } (contraction_{mode} = isometry\ and\ isometric\_relaxation) \\ \\ \dfrac{k_{S_{vis}} \cdot \left(\phi_\chi - alp_s \cdot (w - v)^2\right) - \alpha_1 \cdot \beta_1 \cdot e^{\alpha_1 \cdot (l_2 - l_1)} \cdot (w - v) - \alpha_2 \cdot \beta_2 \cdot e^{\alpha_2 \cdot l_2} \cdot w}{k_{S_{vis}}}, \\ \text{if } (contraction_{mode} = isotony) \\ \\ \phi_\chi - alp_s \cdot (w - v)^2 - \\ - \dfrac{\alpha_1 \cdot \beta_1 \cdot e^{\alpha_1 \cdot (l_2 - l_1)} \cdot (w - v) + \alpha_2 \cdot \beta_2 \cdot e^{\alpha_2 \cdot l_2} \cdot w - \alpha_3 \cdot \beta_3 \cdot e^{\alpha_3 \cdot l_3} \cdot (V_{phys\_rel} - w)}{k_{S_{vis}}}, \\ \text{if } (contraction_{mode} = physiological\_relaxation) \end{cases}$$

$$v_1 = \frac{v_{max}}{10}$$

$$\gamma_2 = \frac{a \cdot d_h \cdot \left(\frac{v_1}{v_{max}}\right)^2}{3 \cdot a \cdot d_h - \frac{(a+1) \cdot v_1}{v_{max}}}$$

$$P_{star} = \begin{cases} \dfrac{a \cdot \left(1 + \frac{v}{v_{max}}\right)}{a - \frac{v}{v_{max}}} & \text{if } v \leq 0 \\ 1 + d_h - \dfrac{d_h^2 \cdot a}{\frac{a \cdot d_h}{\gamma_2} \cdot \left(\frac{v}{v_{max}}\right)^2 + \frac{(a+1) \cdot v}{v_{max}} + a \cdot d_h} & \text{otherwise} \end{cases}$$

$$G_{star} = \begin{cases} 1 + \frac{0.6 \cdot v}{v_{max}} & \text{if } (v \leq 0) \\ \dfrac{P_{star}}{\frac{(0.4 \cdot a + 1) \cdot v}{a \cdot v_{max}} + 1} & \text{if } (0 < v) \text{ and } (v \leq v_1) \\ \dfrac{P_{star} \cdot e^{-\alpha_G \cdot \left(\frac{v - v_1}{v_{max}}\right)^{\alpha_P}}}{\frac{(0.4 \cdot a + 1) \cdot v}{a \cdot v_{max}} + 1} & \text{otherwise} \end{cases}$$

$$case_1 = \frac{a \cdot (0.4 + 0.4 \cdot a)}{v_{max} \cdot ((a+1) \cdot 0.4)^2}$$

$$case_2 = \frac{a \cdot 1 \cdot \left(1 + 0.4 \cdot a + \frac{1.2 \cdot v}{v_{max}} + 0.6 \cdot \left(\frac{v}{v_{max}}\right)^2\right)}{v_{max} \cdot \left(\left(a - \frac{v}{v_{max}}\right) \cdot \left(1 + \frac{0.6 \cdot v}{v_{max}}\right)\right)^2}$$

$$case_3 = \frac{0.4 \cdot a + 1}{a \cdot v_{max}}$$

$$case_4 = \frac{1}{v_{max}} \cdot e^{-\alpha_G \cdot \left(\frac{v - v_1}{v_{max}}\right)^{\alpha_P}} \cdot \left(\frac{0.4 \cdot a + 1}{a} + \alpha_G \cdot \alpha_P \cdot \left(1 + \frac{(0.4 \cdot a + 1) \cdot v}{a \cdot v_{max}}\right) \cdot \left(\frac{v - v_1}{v_{max}}\right)^{\alpha_P - 1}\right)$$

$$p_{prime_v} = \begin{cases} case_1 & \text{if } v \leq -v_{max} \\ case_2 & \text{if } (-v_{max} < v) \text{ and } (v \leq 0) \\ case_3 & \text{if } (0 < v) \text{ and } (v \leq v_1) \\ case_4 & \text{otherwise} \end{cases}$$

$$p_v = \frac{P_{star}}{G_{star}}$$

$$M_A = \frac{\left(\frac{CaTnC}{TnC_{tot}}\right)^{\mu} \cdot \left(1 + k_{\mu}^{\mu}\right)}{\left(\frac{CaTnC}{TnC_{tot}}\right)^{\mu} + k_{\mu}^{\mu}}$$

$$temp_{n1} = (g_1 \cdot l_1 + g_2) \cdot \left(n1_A + \frac{n1_K - n1_A}{\left(n1_C + n1_Q \cdot e^{-n1_B \cdot l_1}\right)^{\frac{1}{n1_\nu}}}\right)$$

$$n_1 = \begin{cases} 0 & \text{if } temp_{n1} < 0 \\ temp_{n1} & \text{if } temp_{n1} < 1 \\ 1 & \text{otherwise} \end{cases}$$

$$L_{oz} = \begin{cases} \dfrac{l_1 + S_0}{S_{046} + S_0} & \text{if } l_1 \leq S_{055} \\ \dfrac{S_0 + S_{055}}{S_{046} + S_0} & \text{otherwise} \end{cases}$$

$$\kappa = \begin{cases} \kappa_1 + \kappa_2 \cdot \frac{v}{v_{max}} & \text{if } v < 0 \\ \kappa_1 & \text{otherwise} \end{cases}$$

$$v_{st} = x_{st} \cdot v_{max}$$

$$q_v = \begin{cases} q_1 - \dfrac{q_2 \cdot v}{v_{max}} & \text{if } v \leq 0 \\ \dfrac{(q_4 - q_3) \cdot v}{v_{st}} + q_3 & \text{if } \ (v \leq v_{st}) \text{ and } \ (0 < v) \\ \dfrac{q_4}{\left(1 + \dfrac{\beta_Q \cdot (v - v_{st})}{v_{max}}\right)^{\alpha_Q}} & \text{otherwise} \end{cases}$$

$$k_{p_v} = \kappa \cdot \kappa_0 \cdot q_v \cdot m_0 \cdot G_{star}$$

$$k_{m_v} = \kappa_0 \cdot q_v \cdot (1 - \kappa \cdot m_0 \cdot G_{star})$$

$$K_\kappa = k_{p_v} \cdot M_A \cdot n_1 \cdot L_{oz} \cdot (1 - N) - k_{m_v} \cdot N$$

$$\frac{\mathrm{d}N}{\mathrm{d}time} = K_\kappa$$

II. SIMULATION OF ACUTE ISCHEMIA

Constants

	Definition	Value	Unit
g_{gap}	the maximal channel conductance	3.9	nS
$K^+_{o,norm}$	the physiological extracellular $[K^+]_o$ concentration	5.4	mM
h	the Hill coefficient	2.2	dimensionless
n	the sensitivity coefficient of i_{KATP}	0.24	dimensionless

Simulation of hyperkalemia

	Definition	0 min	5 min	10 min	15 min
$[K^+]_o$	extracellular K^+ concentration (mM)	5.4	6.2	8.0	9.4

Simulation of anoxia

	Definition	0 min	5 min	10 min	15 min
$[ATP]_i$	intracellular ATP concentration (mM)	6.8	6.0	5	4.5
$k_{0.5}$	the half-maximal channel activation (mM)	0.042	0.117	0.212	0.259

i_{KATP}, ATP-dependent K^+ current.
P_{ATP}, fraction of open ATP-dependent K^+ channels

ATP-dependent K^+ current

$$i_{KATP} = g_{gap} \cdot P_{ATP} \cdot (\frac{[K_o^+]}{[K^+]_{o,norm}})^n \cdot (V - E_K)$$

$$P_{ATP} = \frac{1}{1 + (\frac{[ATP_i]}{k_{0.5}})^h}$$

III. 1D MODEL

This model represents a one-dimensional (1D) strand of coupled cardiomyocytes.
Key Assumptions and Spatial Scales:

- Cardiomyocytes are treated as isopotential points, allowing the strand to be modeled as a continuous 1D medium.
- The electrical and mechanical activity of an individual cell is described by the TP+M model (see Section I).
- The formulation in the 1D model operates in two interconnected geometrical spaces:
  - **Microspace:** The dynamically changing geometry of individual cardiomyocytes.
  - **Macrospace:** The overall geometry of the deforming strand.
- A defined relationship between these spaces captures the bidirectional coupling between electrical excitation and mechanical contraction across tissue and cellular levels.

Strand Geometry and Coordinate System (Figure S2):
We consider a strand with a slack length L_0, where the spatial coordinate x runs from the fixed left boundary ($x = 0$) to the right boundary ($x_F = L_0$). The strand is assumed to consist of identical cardiomyocytes at a sarcomere slack length of 1.67 µm.

Each material point is assigned a fixed Lagrangian coordinate x, defined as its distance from the left end when the strand is in its unstretched and unexcited state. This coordinate x serves as a permanent label for the point, independent of its subsequent displacements during the strand's contractile cycle. An excitation wave propagate from the left strand end ($x = 0$) towards the right end ($x = x_F$), triggering contraction of the strand.

Cellular mechanics (microlevel)

The rheological scheme for a single cardiomyocyte at point x (referred to as cell x) is shown in Figure S2.
$l_1(x, t)$ is the relative change in the sarcomere (CE_x) length of the cell x against its slack length (normalized by the sarcomere slack length of 1.67 µm).
$l(x, t)$ is a relative change in the cell x length per sarcomere (normalized by its sarcomere slack length). In correspondence with the rheological scheme, $l(x, t)$ coincides with the deviation of the length of the parallel elastic element PE_x from its slack length.

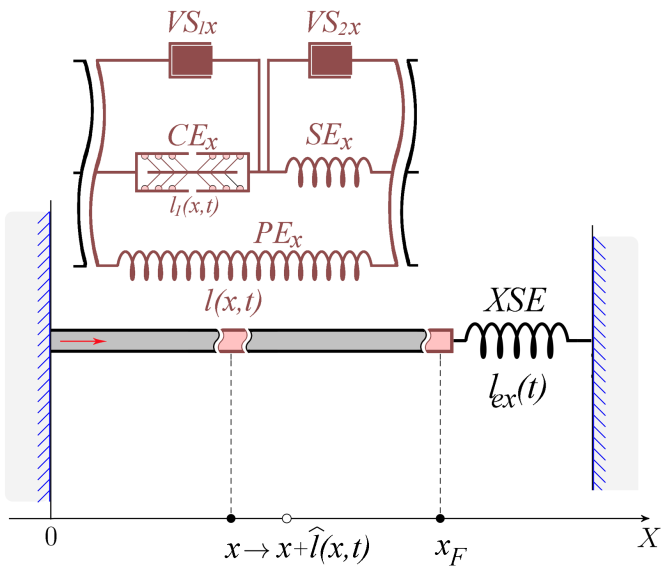

Figure S2: Structure of a 1D heart muscle strand and rheological scheme of car-diomyocytes in which the contractile element (CE_x) connected with in-series and parallel passive elastic elements (PE_x, SE_x). Viscous elements (VS_{1x},VS_{2x}) are in parallel to elements CE_x and SE_x. XSE is the external in-series elastic ele-ment. Variables $l(x, t)$, $l_1(x, t)$ and $l_{ex}(t)$ define deformations of PE_x, CE_x and XSE, respectively, relative to their slack lengths

The following equations define force F_x that cardiomyocyte at point x develops accordingly to the rheological scheme:

$$F_x = F_{CEx} + F_{PEx} + F_{VSx1},$$
$$F_{CEx} = F_{SEx},$$
$$F_{VSx1} = F_{VSx2}.$$

Strand mechanics (macrolevel)

The current time-dependent position of cell x at a given moment during the contractile cycle is $x + \hat{l}(x, t)$, where $\hat{l}(x, t)$ is a displacement of cell x from its reference position in the unstretched and unexcited strand (Fig. S2).

$l_{ex}(t)$ is a deviation of an external serial elastic element XSE from its slack length.

In a series connection, the force must be uniform along the strand. Therefore, the force F_x generated by every cell x is the same and equals the force of XSE:

$$F_x = F_{XSE}.$$

In the isometric mode, the strand length remains fixed. Let $l_m(t)$ be the strand extension from slack length, determined by initial preload ρ and constant during contraction. The displacement of the strand's right end x_F is balanced by stretching the external element XSE, keeping total deformation constant:

$$l_m(t) = l_m(0) = \hat{l}(x_F, t) + l_{ex}(t).$$

Thus, dynamics of $\hat{l}(x,t)$ and $l_{ex}(t)$ describe the macroscopic mechanics of the strand.

Micro- and macromechanics coupling

A key feature of our continuous model is coupling global strand deformations with local cell geometry. We define the displacement $\hat{l}(x,t)$ of point x from its reference position as the integral of relative length changes in cells over segment $[0,x]$ at time t:

$$\hat{l}(x,t) = \int_0^x l(\xi, t) d\xi.$$

In other words, the local deformation of the strand at point x in the macrospace is equal to the relative deformation of cell x in the microspace:

$$\frac{\partial \hat{l}(x,t)}{\partial x} = l(x,t).$$

The above equations govern the coupling between micro- and macromechanics in the model.

Thus, during the propagation of the electrical signal from the left to the right end of the strand lengths of all contracting cells continuously change, providing for the global deformation of the strand and overall force generation.

The boundary conditions (at $x = 0$ and $x = x_F$) during isometric mode are:

$$\hat{l}(0,t) = 0,$$
$$\hat{l}(x_F, t) + l_{ex}(t) = \hat{l}(x_F, 0) + l_{ex}(0).$$

The initial conditions ($t = 0$) for $\hat{l}(x,t)$ and $l_{ex}(t)$ are determined by force balance when a preload ρ is applied, stretching the strand from its slack length on $l_m(0)$.

Micro- and macroelectrical coupling

The electrical excitation of the strand is governed by the cable reaction-diffusion equation for the membrane potential $V(x,t)$:

$$\frac{\partial V(x,t)}{\partial t} = D \cdot \frac{\partial^2 V(x,t)}{\partial x^2} - \frac{1}{C_m(x)} \cdot \sum i_{\text{ion}}(x,t),$$

where $C_m(x)$ is the membrane capacitance of cell x, $\sum i_{\text{ion}}(x,t)$ represents all local transmembrane ionic currents in the cell x, and D is the electrodiffusion coefficient.

The boundary conditions at $x = 0$:

$$D \frac{\partial V(0,t)}{\partial x} = -\frac{1}{C_m} i_{stim}(t);$$

at $x = x_F$:

$$\frac{\partial V(x_F, t)}{\partial x} = 0.$$

A resting potential value is used for all strand cells as initial conditions at $t = 0$, which is the same as in the TP+M model (see Section I):

$$V(x, 0) = V_{init}(x).$$
